# Supplementary material for: Anionic ring-opening polymerization of functional epoxide monomers in the solid state
Source: Nat Commun. 2023 Sep 20;14:5855. doi: 10.1038/s41467-023-41576-0 (PMC10511433; doi:10.1038/s41467-023-41576-0)
Supplement: Supplementary file 1 — Supplementary Information [file 41467_2023_41576_MOESM1_ESM.pdf]

Supplementary Information

Anionic Ring-Opening Polymerization of Functional Epoxide Monomers in the Solid State

*Jihye Park,[1] Ahyun Kim,[1] and Byeong-Su Kim[1]**

[1]Department of Chemistry, Yonsei University, Seoul 03722, Republic of Korea

E-mail: bskim19@yonsei.ac.kr

Table of Contents

List of Figures

Supplementary Fig. 1. Library of investigated epoxide monomers. 6

Supplementary Fig. 2. ^{1}H NMR spectrum of MPG (400 MHz, $CDCl_3$). 7

Supplementary Fig. 3. ^{13}C NMR spectrum of MPG (101 MHz, $CDCl_3$). 8

Supplementary Fig. 4. ^{1}H–^{1}H COSY NMR spectrum of MPG in $CDCl_3$. 9

Supplementary Fig. 5. ^{1}H–^{13}C HSQC NMR spectrum of MPG in $CDCl_3$. 10

Supplementary Fig. 6. ^{1}H NMR spectrum of DPG (400 MHz, $CDCl_3$). 11

Supplementary Fig. 7. ^{13}C NMR spectrum of DPG (101 MHz, $CDCl_3$). 12

Supplementary Fig. 8. ^{1}H–^{1}H COSY NMR spectrum of DPG in $CDCl_3$. 13

Supplementary Fig. 9. ^{1}H–^{13}C HSQC NMR spectrum of DPG in $CDCl_3$. 14

Supplementary Fig. 10. ^{1}H NMR spectrum of BPG (400 MHz, $CDCl_3$). 15

Supplementary Fig. 11. ^{13}C NMR spectrum of BPG (101 MHz, $CDCl_3$). 16

Supplementary Fig. 12. ^{1}H–^{1}H COSY NMR spectrum of BPG in $CDCl_3$. 17

Supplementary Fig. 13. ^{1}H–^{13}C HSQC NMR spectrum of BPG in $CDCl_3$. 18

Supplementary Fig. 14. ^{1}H NMR spectrum of TGE (400 MHz, $CDCl_3$). 19

Supplementary Fig. 15. ^{13}C NMR spectrum of TGE (101 MHz, $CDCl_3$). 20

Supplementary Fig. 16. ^{1}H–^{1}H COSY NMR spectrum of TGE in $CDCl_3$. 21

Supplementary Fig. 17. ^{1}H–^{13}C HSQC NMR spectrum of TGE in $CDCl_3$. 22

Supplementary Fig. 18. ^{1}H NMR spectrum of (*s*)-TGE (400 MHz, $CDCl_3$). 23

Supplementary Fig. 19. ^{13}C NMR spectrum of (*s*)-TGE (101 MHz, $CDCl_3$). 24

Supplementary Fig. 20. ^{1}H–^{1}H COSY NMR spectrum of (*s*)-TGE in $CDCl_3$. 25

Supplementary Fig. 21. ^{1}H–^{13}C HSQC NMR spectrum of (*s*)-TGE in $CDCl_3$. 26

Supplementary Fig. 22. DSC thermogram of MPG. 27

Supplementary Fig. 23. DSC thermogram of DPG. 28

Supplementary Fig. 24. DSC thermogram of BPG. 29

Supplementary Fig. 25. DSC thermogram of TGE. 30

Supplementary Fig. 26. DSC thermogram of (*s*)-TGE. 31

Supplementary Fig. 27. ^{1}H NMR spectrum of (*s*)-PTGE polymer obtained via ball milling AROP for 2 h (400 MHz, CD_2Cl_2) ……………………………………………..………………………...32

Supplementary Fig. 28. ^{13}C NMR spectrum of PMPG (101 MHz, Acetone). ……………………....33

Supplementary Fig. 29. ^{13}C NMR spectrum of PDPG (101 MHz, Acetone). ……………………....34

Supplementary Fig. 30. Solid-state ^{13}C NMR spectrum of PBPG. ……………………………….....35

Supplementary Fig. 31. ^{13}C NMR spectrum of PTGE (201 MHz, CD_2Cl_2). ……………………….. 36

Supplementary Fig. 32. ^{13}C NMR spectrum of (*s*)-PTGE (201 MHz, CD_2Cl_2). ……………………. 37

Supplementary Fig. 33. Assignment of methine peaks in the ^{13}C NMR spectra of (a) atactic PTGE and (b) isotactic (*s*)-PTGE (201 MHz, CD_2Cl_2). ……………………………..…………………………38

Supplementary Fig. 34. ^{1}H NMR spectrum of ball milling AROP of (*s*)-$PTGE_{15}$ in the absence of *t*-BuP_4 base (400 MHz, $CDCl_3$). ……………………………………..…………………………40

Supplementary Fig. 35. Ball milling AROP of (*s*)-$PTGE_{15}$ without benzyl alcohol initiator.
(a) ^{1}H NMR spectrum (400 MHz, $CDCl_3$), (b) GPC trace measured in THF using RI signal with PS standards. ……………………………………………………………..…………………………41

Supplementary Fig. 36. MALDI-ToF MS spectrum of the isolated PMPG polyether with individual peak assignments in the selected region……………………………………..……..……………42

Supplementary Fig. 37. MALDI-ToF MS spectrum of the isolated PDPG polyether with individual peak assignments in the selected region...……..……………………………………………………43

Supplementary Fig. 38. MALDI-ToF MS spectrum of the isolated PBPG polyether with individual peak assignments in the selected region..………………………………..……..……………………44

Supplementary Fig. 39. MALDI-ToF MS spectrum of the isolated PTGE polyether with individual peak assignments in the selected region. ………………………………..……..……………………45

Supplementary Fig. 40. MALDI-ToF MS spectrum of the isolated (*s*)-PTGE polyether with individual peak assignments in the selected region. ………………………………..……..……………………46

Supplementary Fig. 41. Initiation mechanism for the elimination of monomers. ……………………47

Supplementary Fig. 42. Distribution histogram of individual initiating group in the resulting (*s*)-PTGE polyether displayed in Supplementary Fig. 40. ………………………..……..……………………47

Supplementary Fig. 43. GPC curves of (*s*)-PTGE with different polymerization time of 5, 10, 20, and 30 min measured in THF using RI signal with PS standards. …………..……..………………………48

Supplementary Fig. 44. GPC curves of (*s*)-PTGE with different degree of polymerization of 15, 25, 50, 75, and 100 measured in THF using RI signal with PS standards. ……..……..……………………49

Supplementary Fig. 45. Synthesis of (*s*)-PTGE via the conventional solution polymerization. (a) ^{1}H NMR spectra (400 MHz, CDCl3), and (b) GPC trace of (s)-PTGE polymer.. ……..……..……………50

Supplementary Fig. 46. Ball milling degradation of (*s*)-PTGE prepared via solution polymerization.

(a) GPC curves of (s)-PTGE with different degradation times of 1 min (red), 5 min (orange), 10 min (green), 20 min (blue), and 30 min (purple). (b) Changes in Mn measured by GPC as a function of ball milling time for degradation of (s)-PTGE prepared via solution polymerization.. ……..……..……………………………………………………………………………………51

Supplementary Fig. 47. DSC thermograms of the obtained polyethers determined via DSC between –80 and 150 °C at a rate of 10 °C/min. Note that no T_g was observed for $PBPG_{50}$……………………52

Supplementary Fig. 48. DSC thermograms of the obtained PBPG polymer and (*s*)-PTGE polymer determined via DSC between –80 and 250 °C at a rate of 10 °C/min. ………………………………53

Supplementary Fig. 49. ^{1}H NMR spectrum of initial bulk polymerization of MPG monomer (400 MHz, $CDCl_3$) ………………………………………………………………………..……………54

Supplementary Fig. 50. ^{1}H NMR spectrum of initial bulk polymerization of DPG monomer (400 MHz, $CDCl_3$) ………………………………………………………………………..……………55

Supplementary Fig. 51. ^{1}H NMR spectrum of initial bulk polymerization of BPG monomer (400 MHz, $CDCl_3$) ………………………………………………………………………..……………56

Supplementary Fig. 52. ^{1}H NMR spectrum of initial bulk polymerization of TGE monomer (400 MHz, $CDCl_3$) ………………………………………………………………………..……………57

Supplementary Fig. 53. ^{1}H NMR spectrum of initial bulk polymerization of (*s*)-TGE monomer (400 MHz, $CDCl_3$) ……………………………………………………………………..……..58

Supplementary Fig. 54. Type of the dominant reaction mechanism during ball milling polymerization of (a) PMPG, (b) PDPG, (c) PBPG, (d) PTGE, and (e) (*s*)-PTGE. …………………………..……..59

Supplementary Fig. 55. Polymerization kinetics of resulting polyether; First-order kinetic plot of $\ln([M]_0/[M]_t)$ over polymerization time of (a) PMPG, (b) PDPG, (c) PBPG, (d) PTGE, and (e) (*s*)-PTGE……………………………………………………………………………………..60

Supplementary Fig. 56. IR thermometer images showing temperature inside the jar after polymerization for each time at 30 Hz. ……………………………………………………..……..61

Supplementary Fig. 57. Ball milling AROP of liquid benzyl glycidyl ether (BGE) with a target DP of 50. A full conversion was observed in 20 min. (a) ^{1}H NMR spectrum (400 MHz, $CDCl_3$), and (b) GPC trace of the resulting poly(benzyl glycidyl ether) (PBGE). ……………………..…………..……..63

Supplementary Fig. 58. Comparative monomer conversions of the benzyl glycidyl ether (BGE) via polymerization under (a) solid-state ball milling polymerization, and (b) solution polymerization in toluene at 60 °C. ……………………………………………………………………………..……..64

Supplementary Fig. 59. Temperature-controllable ball-milling equipment used in this study; cooling jackets (left), MM400 with cooling jackets (center), and overall mechanochemical polymerization setup with chiller (right). ……………………………………………………………………..……..65

Supplementary Fig. 60. IR thermometer images showing temperature inside the jar after polymerization using (a) Ball-milling using MM400 under ambient condition and (b) temperature-controllable ball-milling MM400. All reactions were performed for 30 min at 30 Hz. ………..……..66

Supplementary Fig. 61. Series of plots of monomer conversion vs. melting point or molecular weight for the various functional epoxide monomers. (a–c) Series of plots of monomer conversion vs. melting point for various functional epoxide monomers at reaction times of (a) 10 min, (b) 20 min, and (c) 30 min, and (d–f) series of plots of monomer conversion vs. molecular weight for the various functional epoxide monomers at reaction times of (d) 10 min, (e) 20 min, and (f) 30 min. All data were collected in triplicate, and the average values were reported with standard deviation.……………..…………..67

List of Tables

Supplementary Table 1. Investigation of ball milling parameters. (a) Effect of type of jar and balls, (b) effect of size of balls, and (c) effect of the number of balls. All polymerization was performed using MPG monomer. ……………………………..……………………………..………………..…...39

Supplementary Table 2. Characterization of the synthesized (*s*)-PTGE polymer with different polymerization time. …………..……..………………………………………………………….…48

Supplementary Table 3. Characterization of the synthesized (*s*)-PTGE polymer with different degree of polymerization. …………..……..……………………………………………………………49

Supplementary Table 4. Comparison of monomer conversions under different experimental setup: ball milling, solution, and bulk polymerization. ……………………………………………..….62

List of Notes

Supplementary Note 1. Mechanochemical polymerization conversion of (a) PMPG and PDPD, (b) PTGE and (*s*)-PTGE. ………………………..……………………………..………………..…...69

Supplementary Note 2. Theoretical molecular weight of polymer. …….….…..………………….....69

Supplementary Note 3. Molecular weight of (a) PMPG and PDPG, (b) PTGE and (*s*)-PTGE calculated from ^{1}H NMR spectrum. ……………………..……………………………..……………...…...69

Supplementary Note 4. Degree of polymerization (DP) of (a) PMPG and PDPG, (b) PTGE and (*s*)-PTGE calculated from ^{1}H NMR spectrum. ……..……………………………..……………...…...69

Supplementary Note 5. Initiation efficiency (IE%) calculated from MALDI-ToF spectrum at specific degree of polymerization (a) BnOH initiation and (b) self-initiation. …………..…………..…...…...69

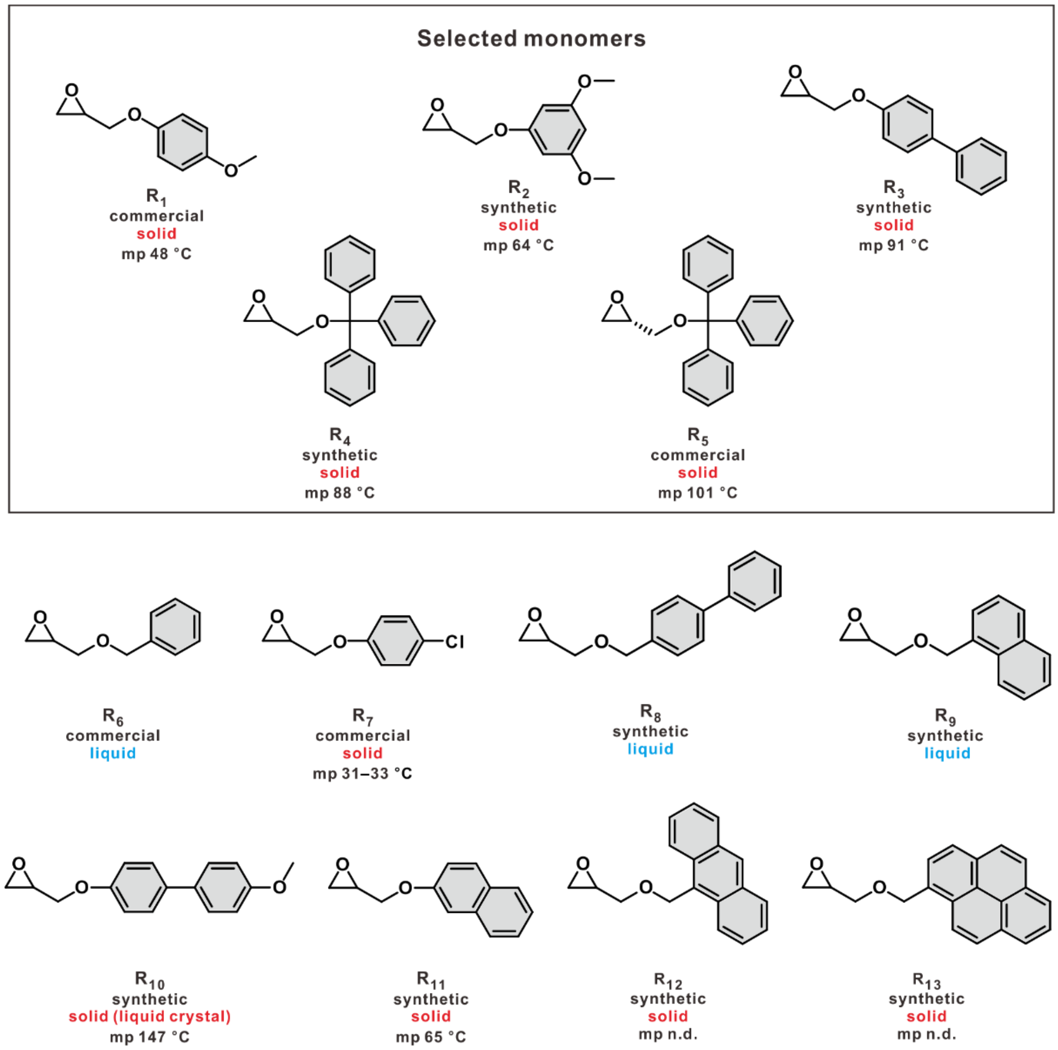

Supplementary Fig. 1. Library of investigated epoxide monomers. Among all 13 monomers, representative five monomers (**R**$_1$–**R**$_5$) were selected, and eight monomers were excluded following reasons: To perform the solid-state mechanochemical polymerization, liquid monomers were not employed (**R**$_6$, **R**$_8$, and **R**$_9$). **R**$_7$ monomer has m.p. of near room temperature, which resulted in difficult handling. A solubility of **R**$_{10}$ monomer and resulting polymer prepared from **R**$_{11}$ monomer were low, resulting in difficult to analyze. Ball milling polymerization of **R**$_{12}$ and **R**$_{13}$ monomers were not conducted possibly due to low purity.

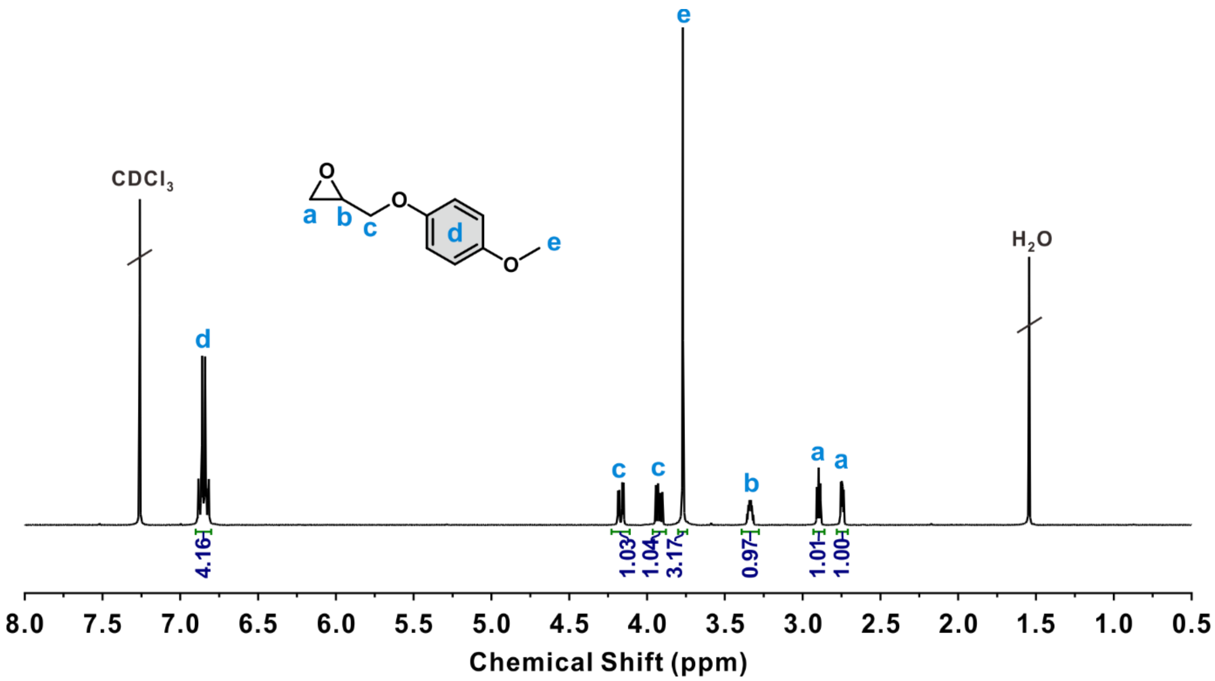

Supplementary Fig. 2. ^{1}H NMR spectrum of MPG (400 MHz, $CDCl_3$).

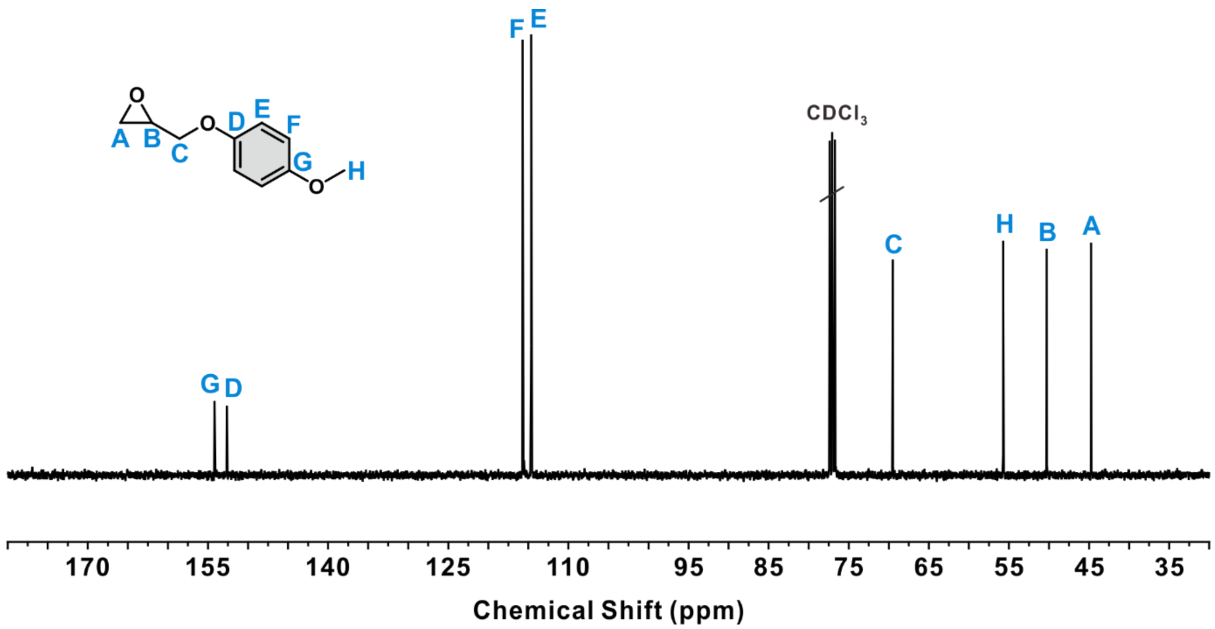

Supplementary Fig. 3. ^{13}C NMR spectrum of MPG (101 MHz, $CDCl_3$).

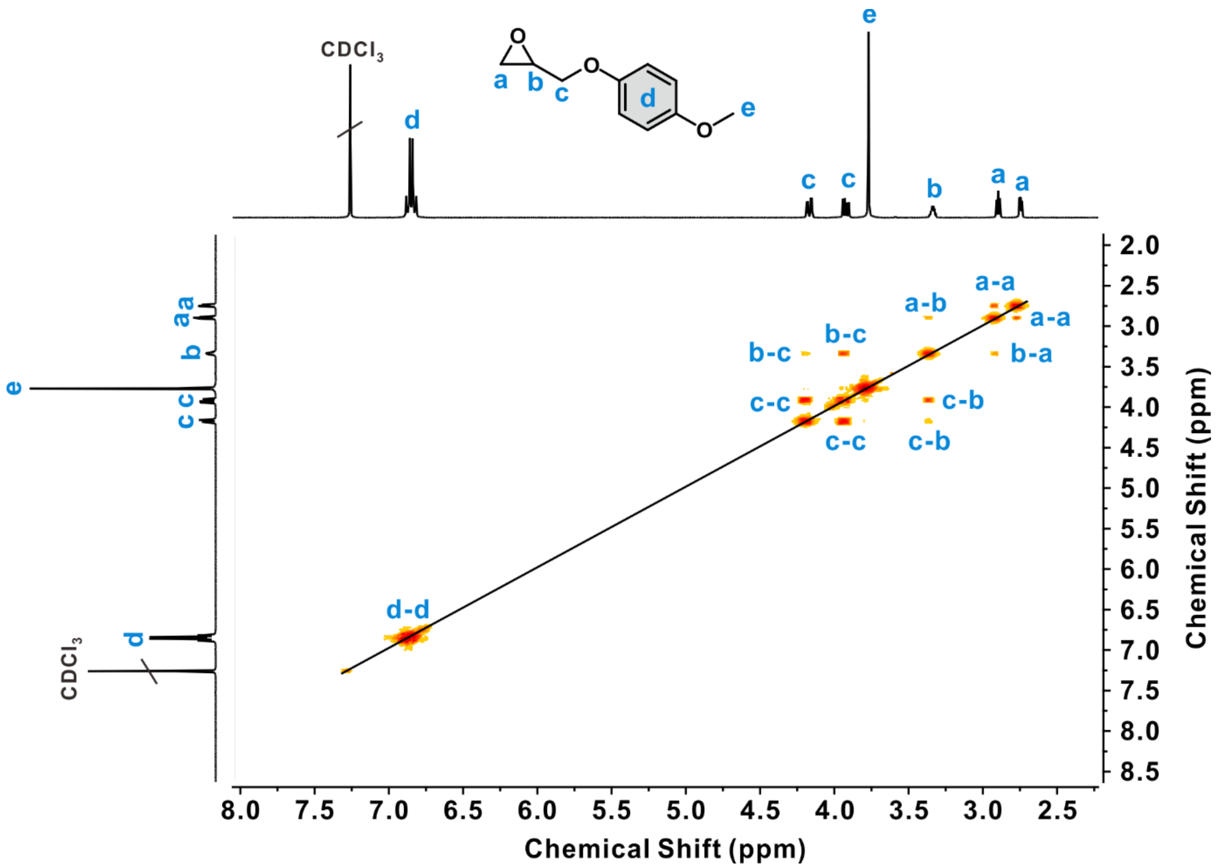

Supplementary Fig. 4. ^{1}H–^{1}H COSY NMR spectrum of MPG in $CDCl_3$.

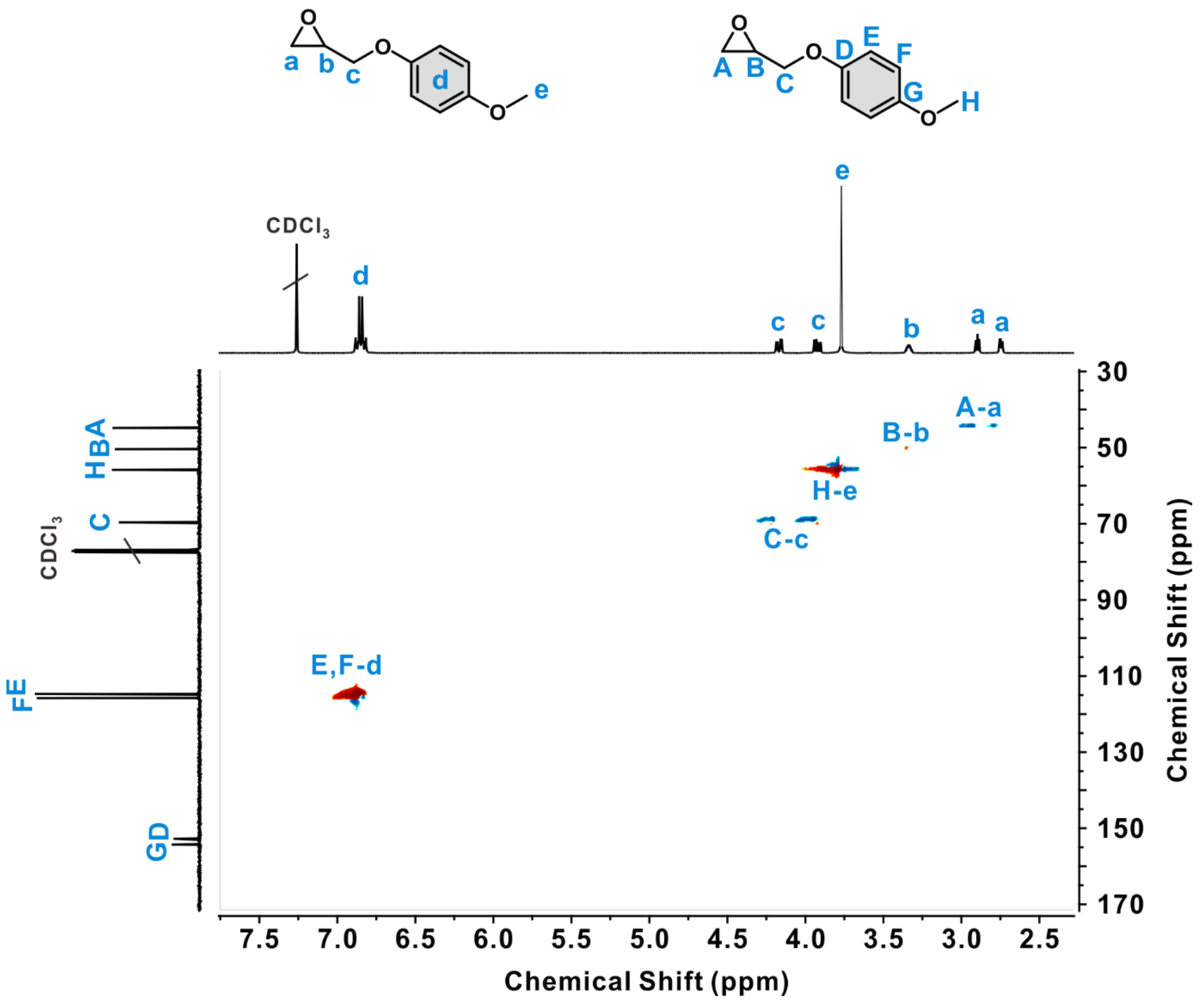

Supplementary Fig. 5. ^1H–^{13}C HSQC NMR spectrum of MPG in $CDCl_3$.

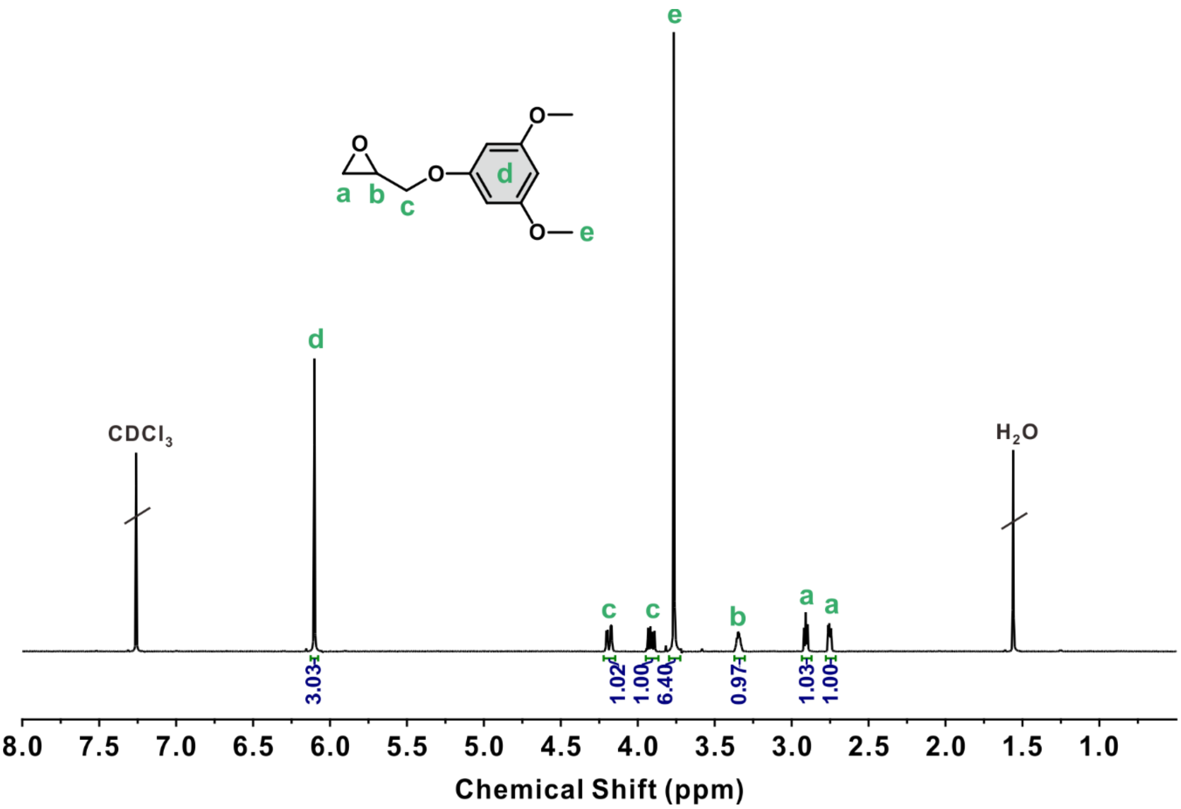

Supplementary Fig. 6. ^{1}H NMR spectrum of DPG (400 MHz, $CDCl_3$).

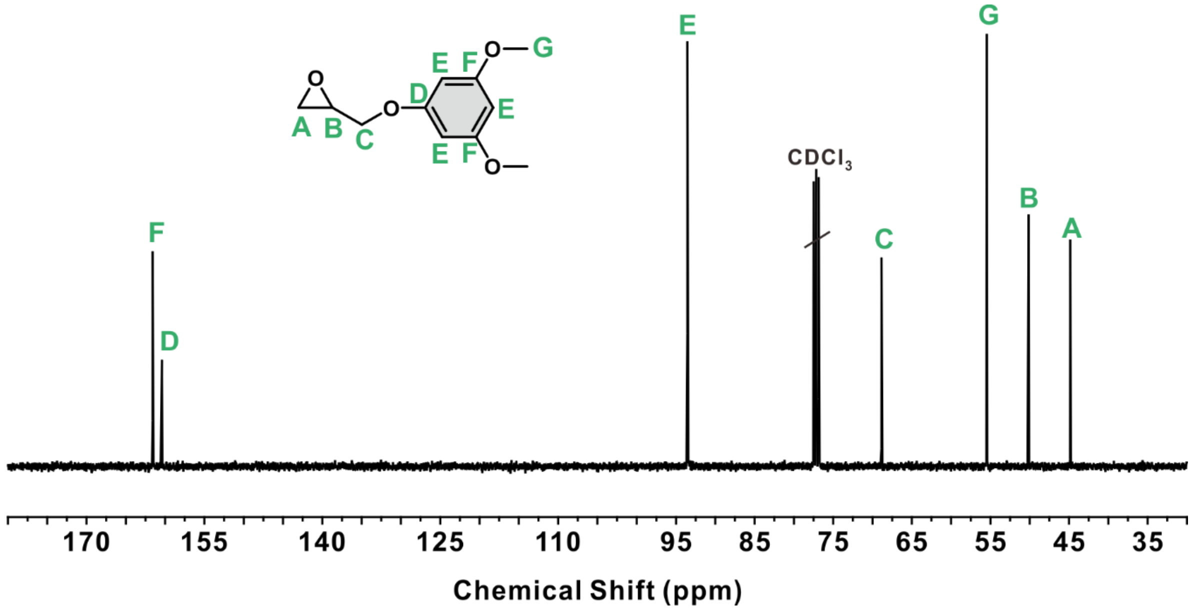

Supplementary Fig. 7. ^{13}C NMR spectrum of DPG (101 MHz, $CDCl_3$).

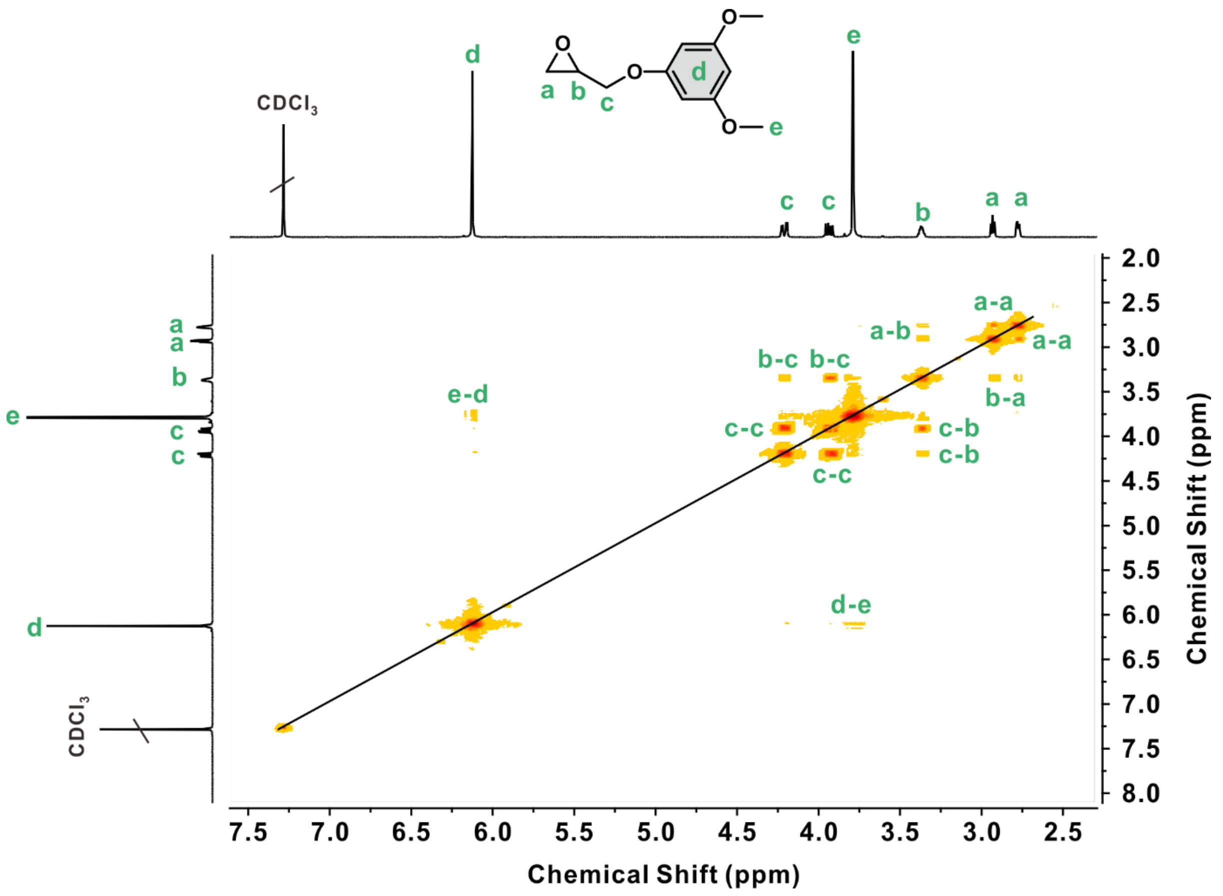

Supplementary Fig. 8. ^{1}H–^{1}H COSY NMR spectrum of DPG in $CDCl_3$.

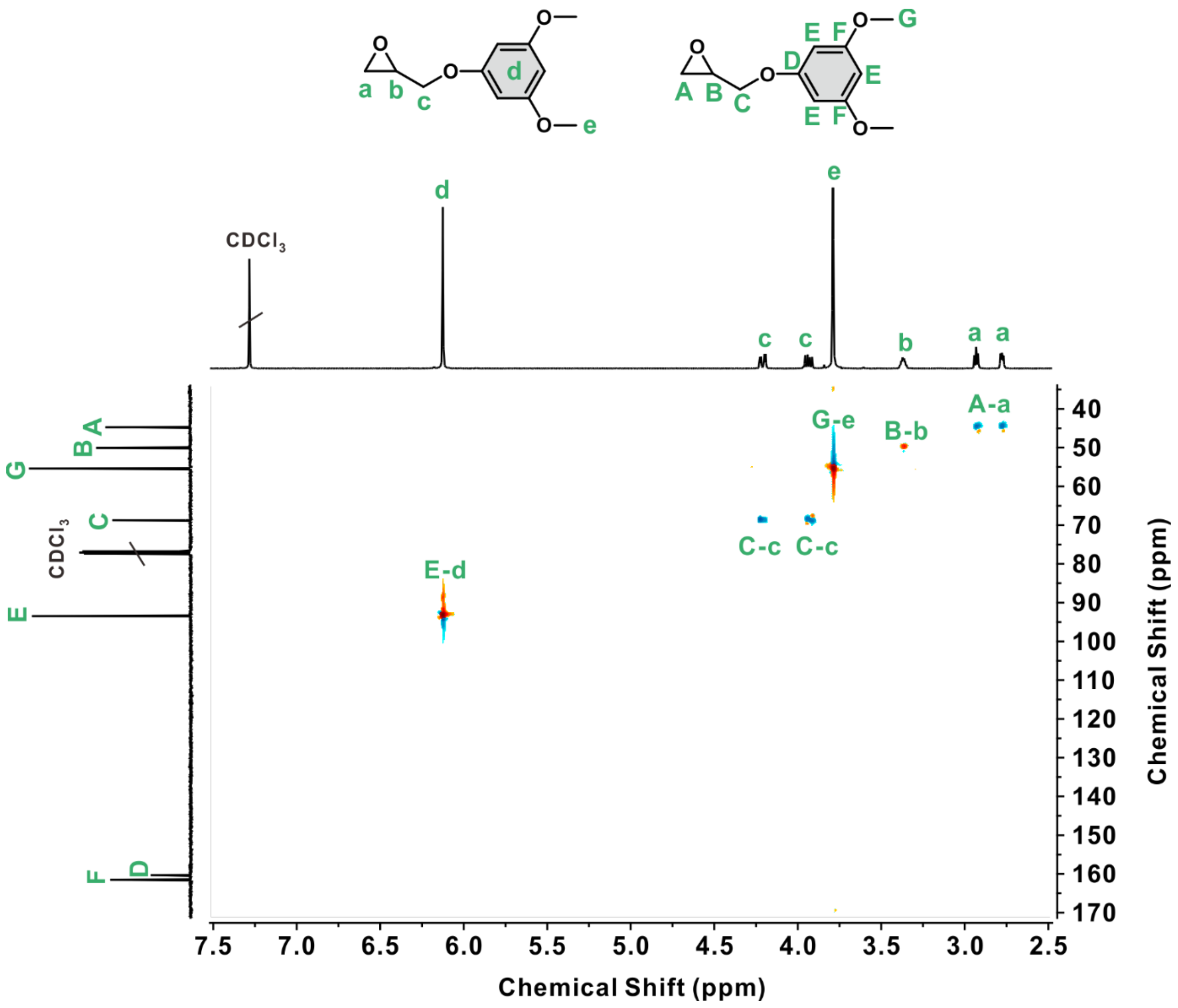

Supplementary Fig. 9. ^{1}H–^{13}C HSQC NMR spectrum of DPG in $CDCl_3$.

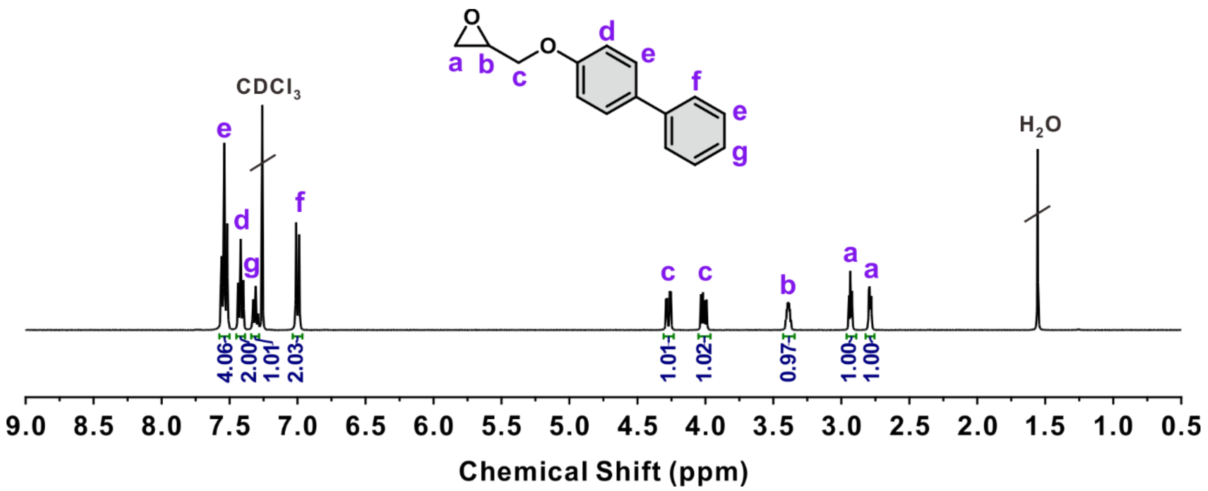

Supplementary Fig. 10. ^{1}H NMR spectrum of BPG (400 MHz, $CDCl_3$).

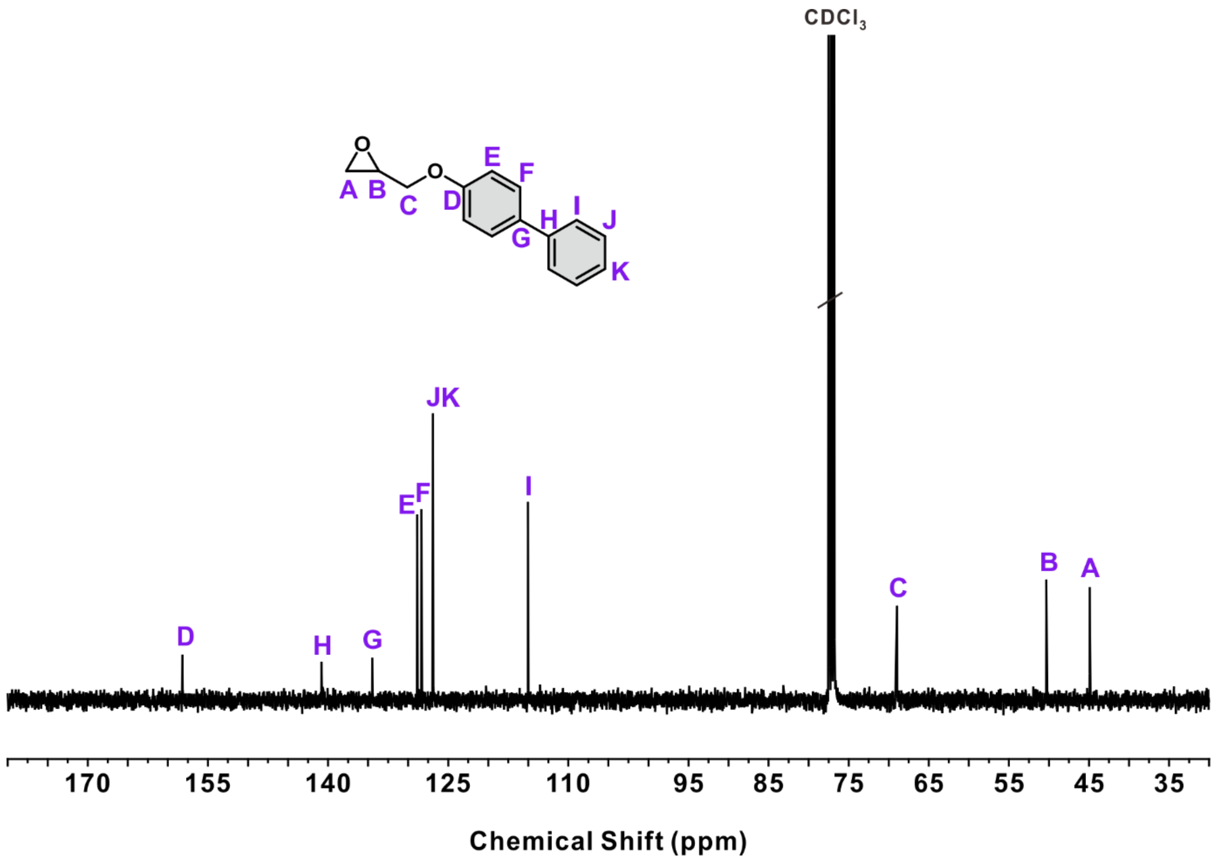

Supplementary Fig. 11. ^{13}C NMR spectrum of BPG (101 MHz, $CDCl_3$).

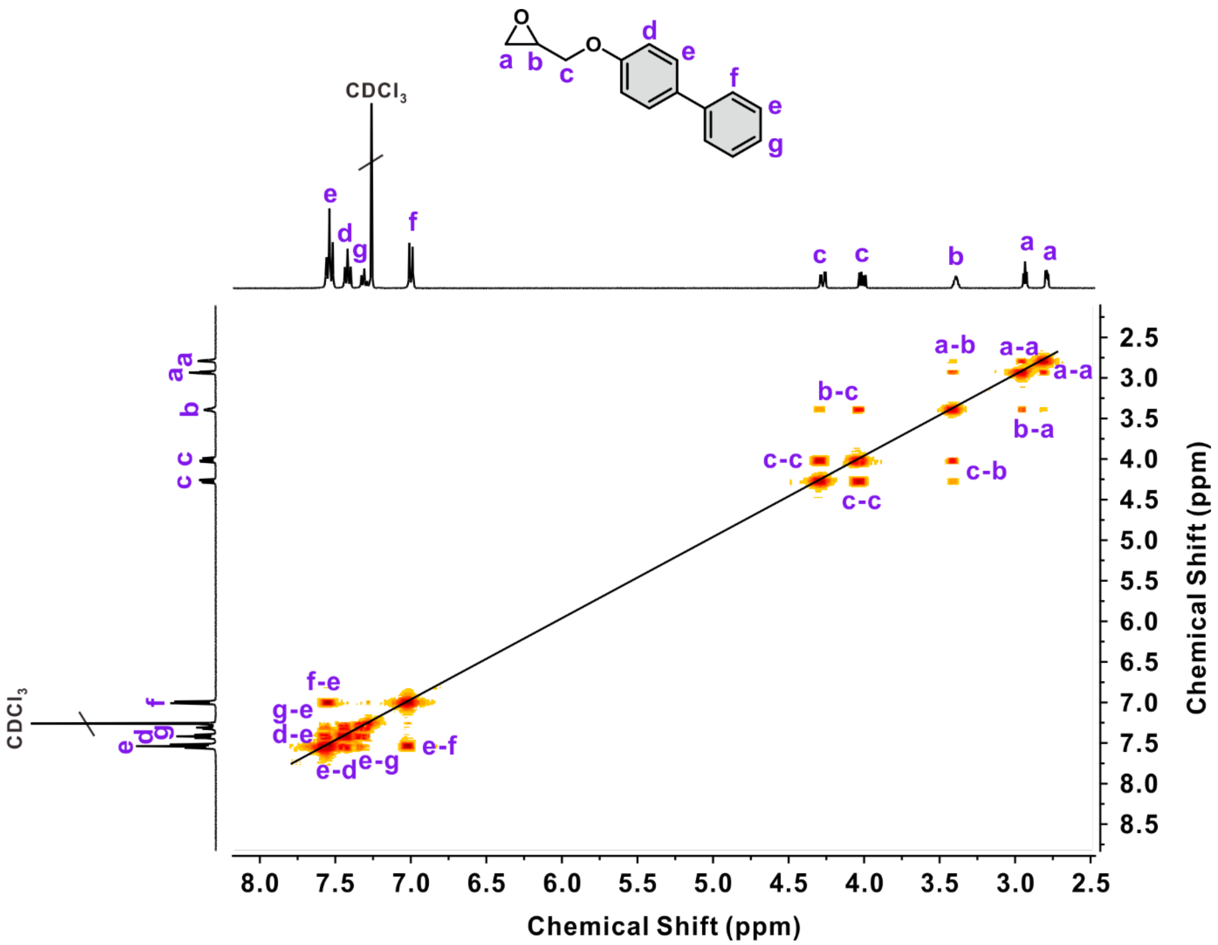

Supplementary Fig. 12. ^{1}H–^{1}H COSY NMR spectrum of BPG in $CDCl_3$.

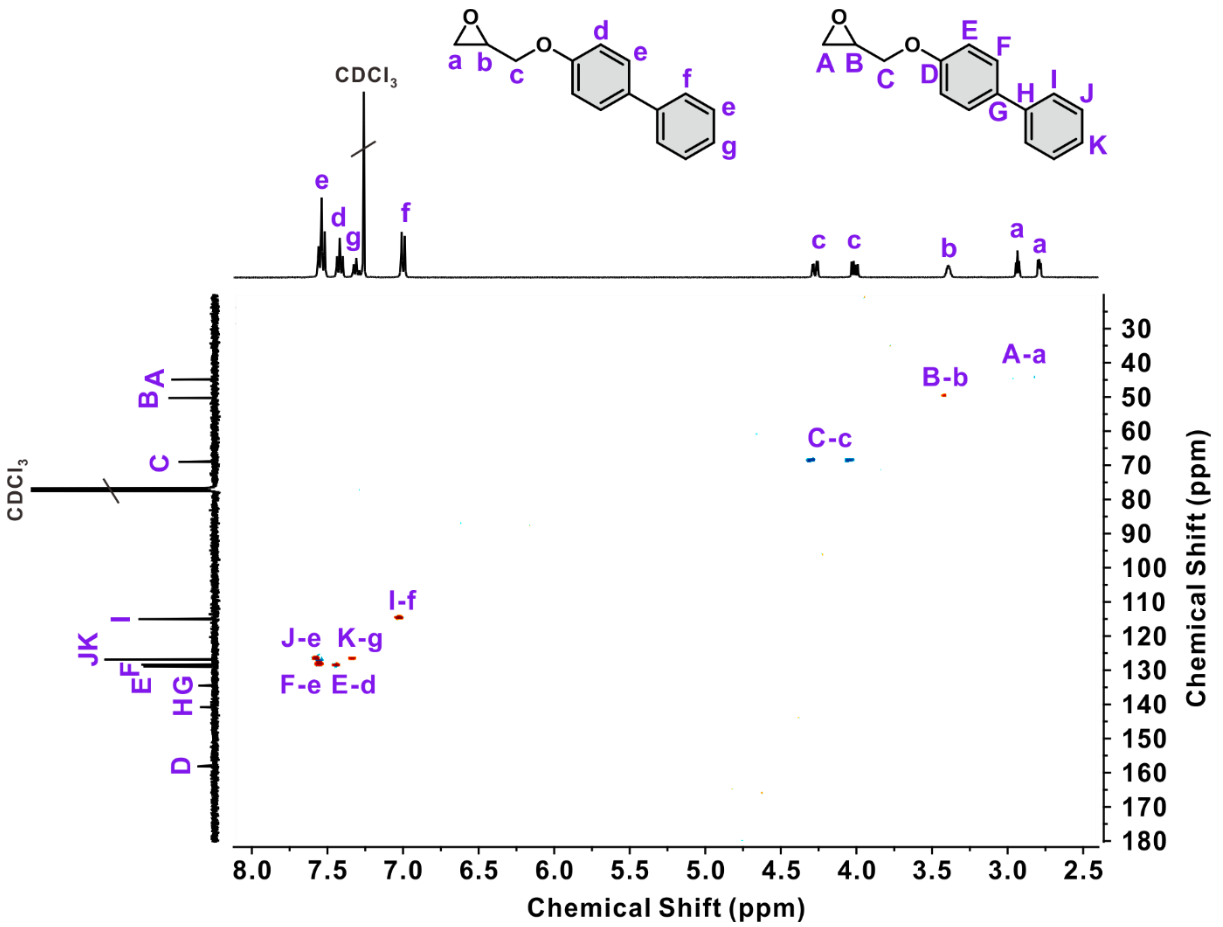

Supplementary Fig. 13. ^{1}H–^{13}C HSQC NMR spectrum of BPG in $CDCl_3$.

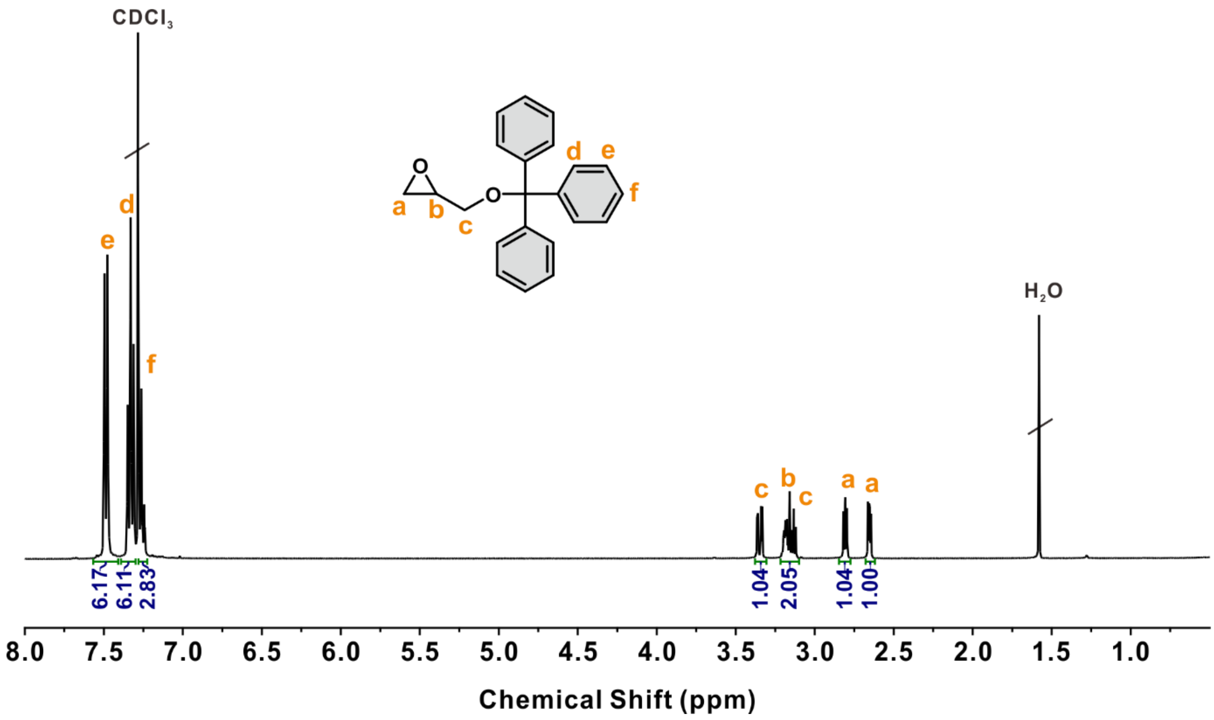

Supplementary Fig. 14. ^{1}H NMR spectrum of TGE (400 MHz, $CDCl_3$).

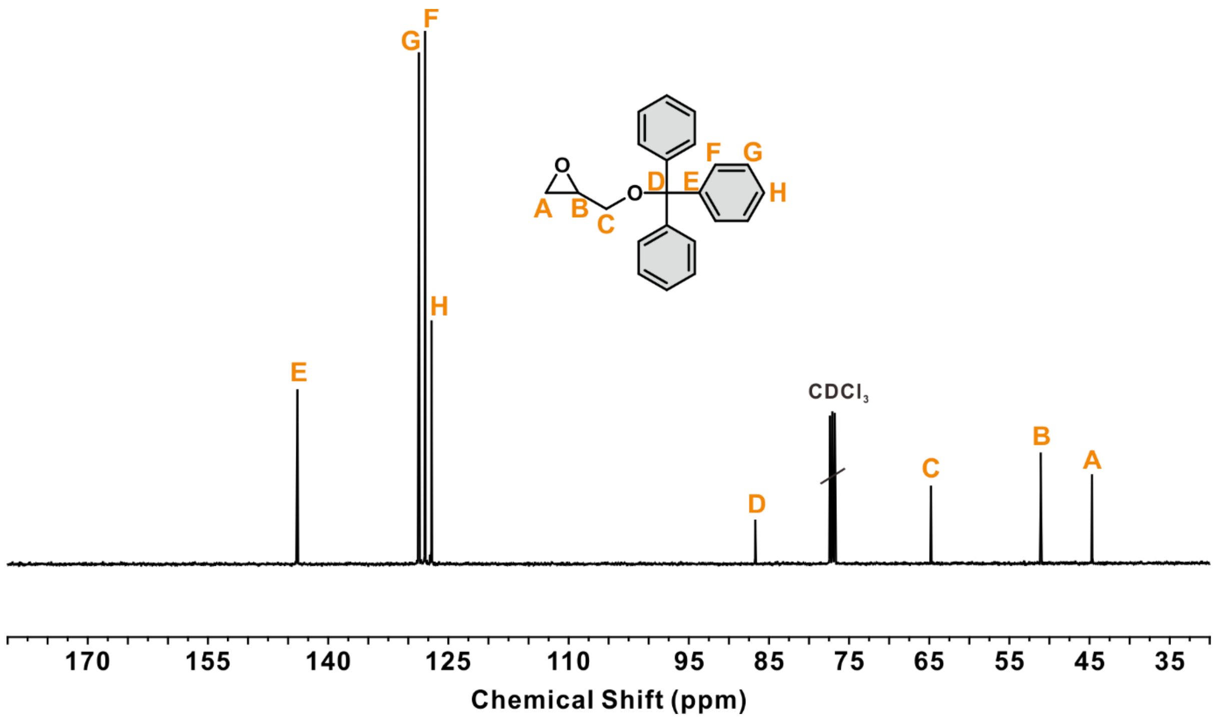

Supplementary Fig. 15. ^{13}C NMR spectrum of TGE (101 MHz, $CDCl_3$).

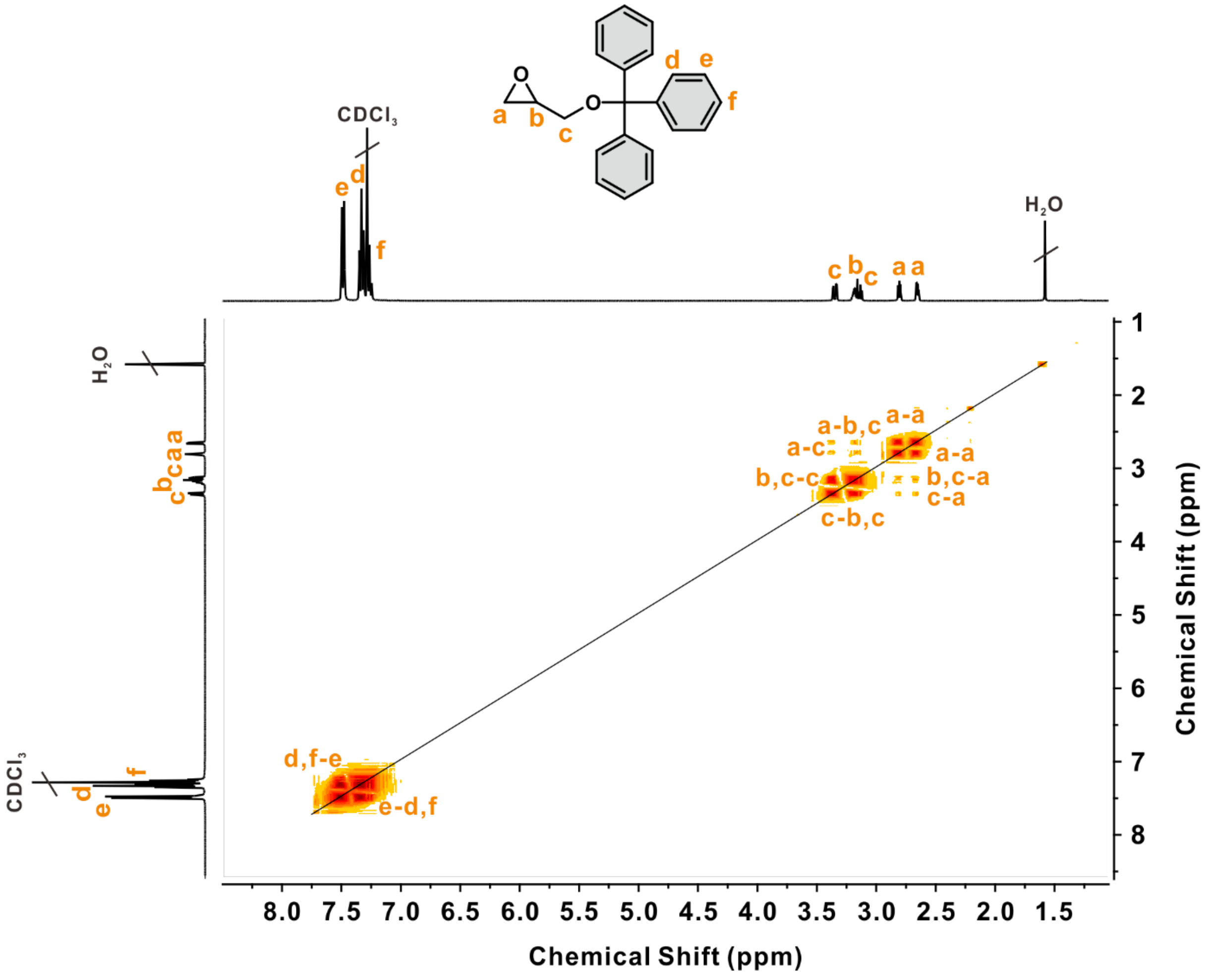

Supplementary Fig. 16. ^1H–^1H COSY NMR spectrum of TGE in $CDCl_3$.

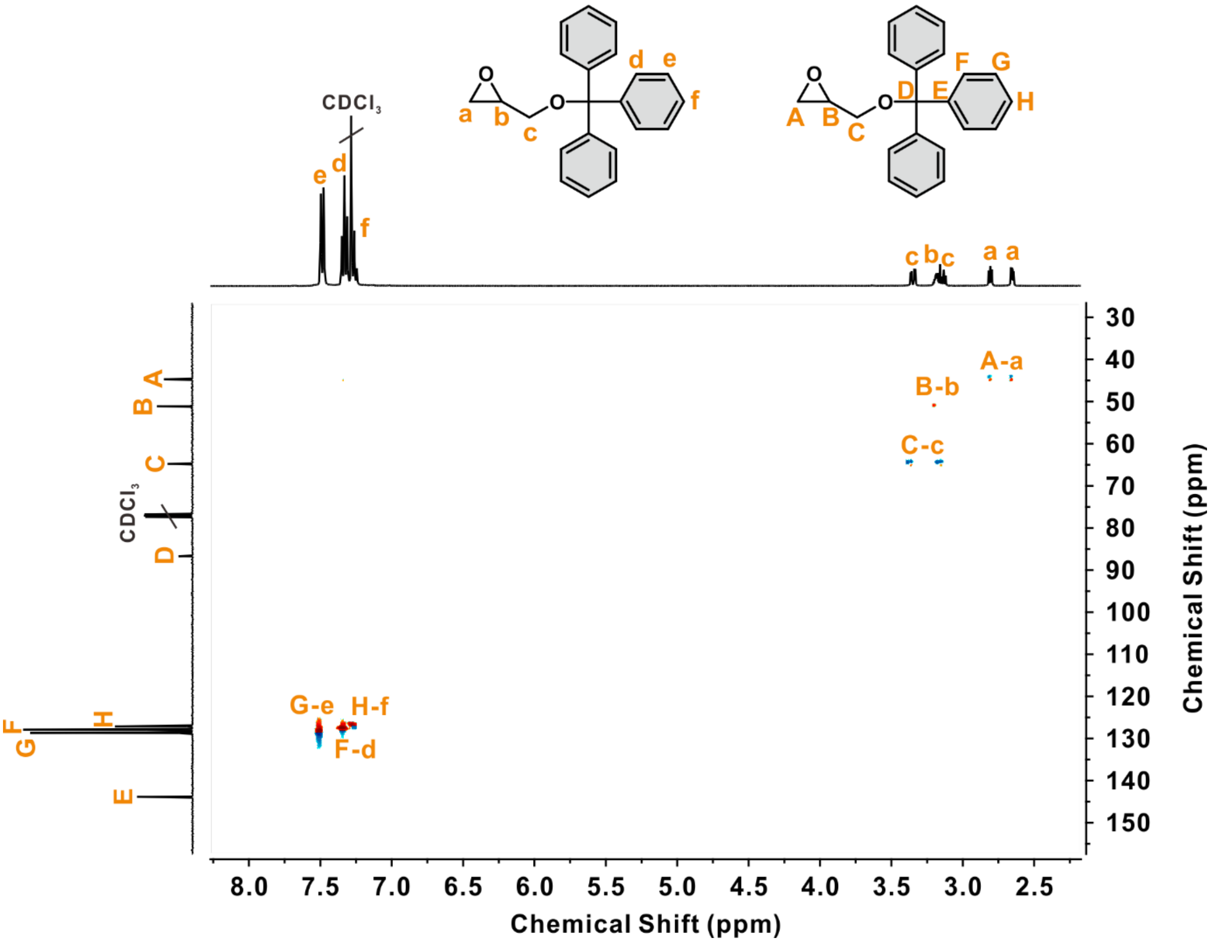

Supplementary Fig. 17. ^{1}H–^{13}C HSQC NMR spectrum of TGE in $CDCl_3$.

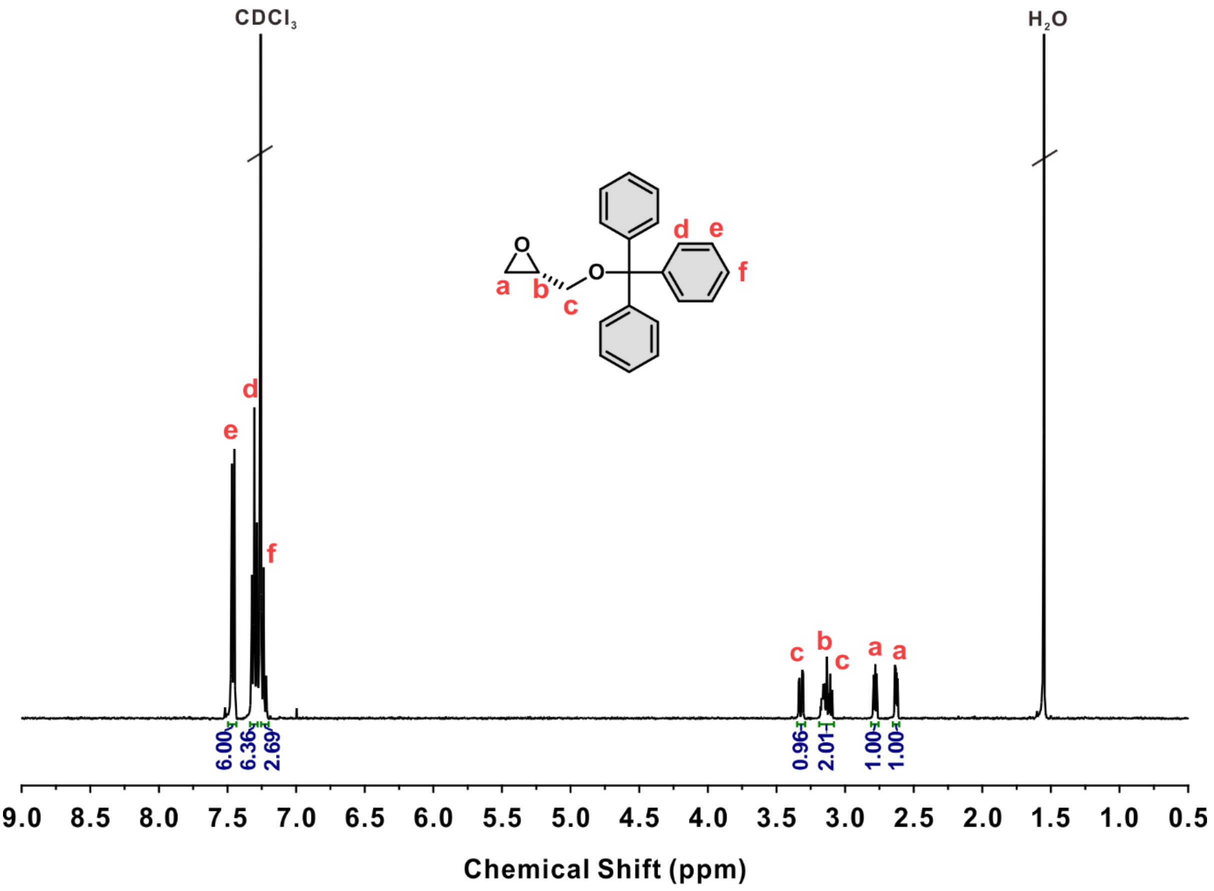

Supplementary Fig. 18. ^{1}H NMR spectrum of (*s*)-TGE (400 MHz, $CDCl_3$).

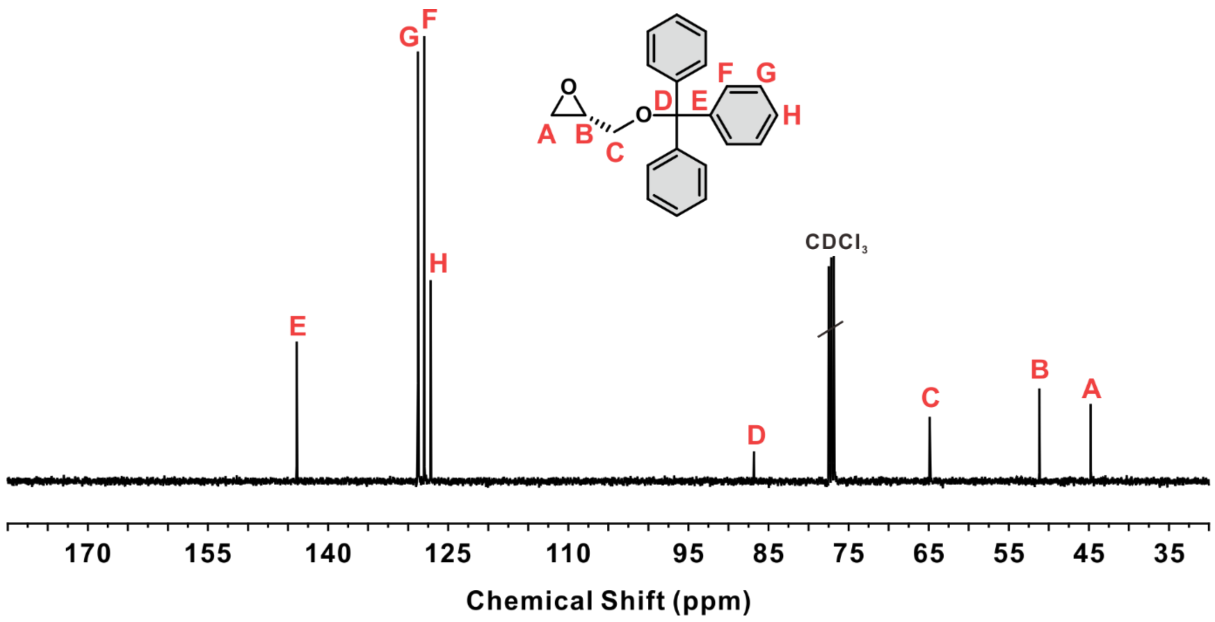

Supplementary Fig. 19. ^{13}C NMR spectrum of (*s*)-TGE (101 MHz, $CDCl_3$).

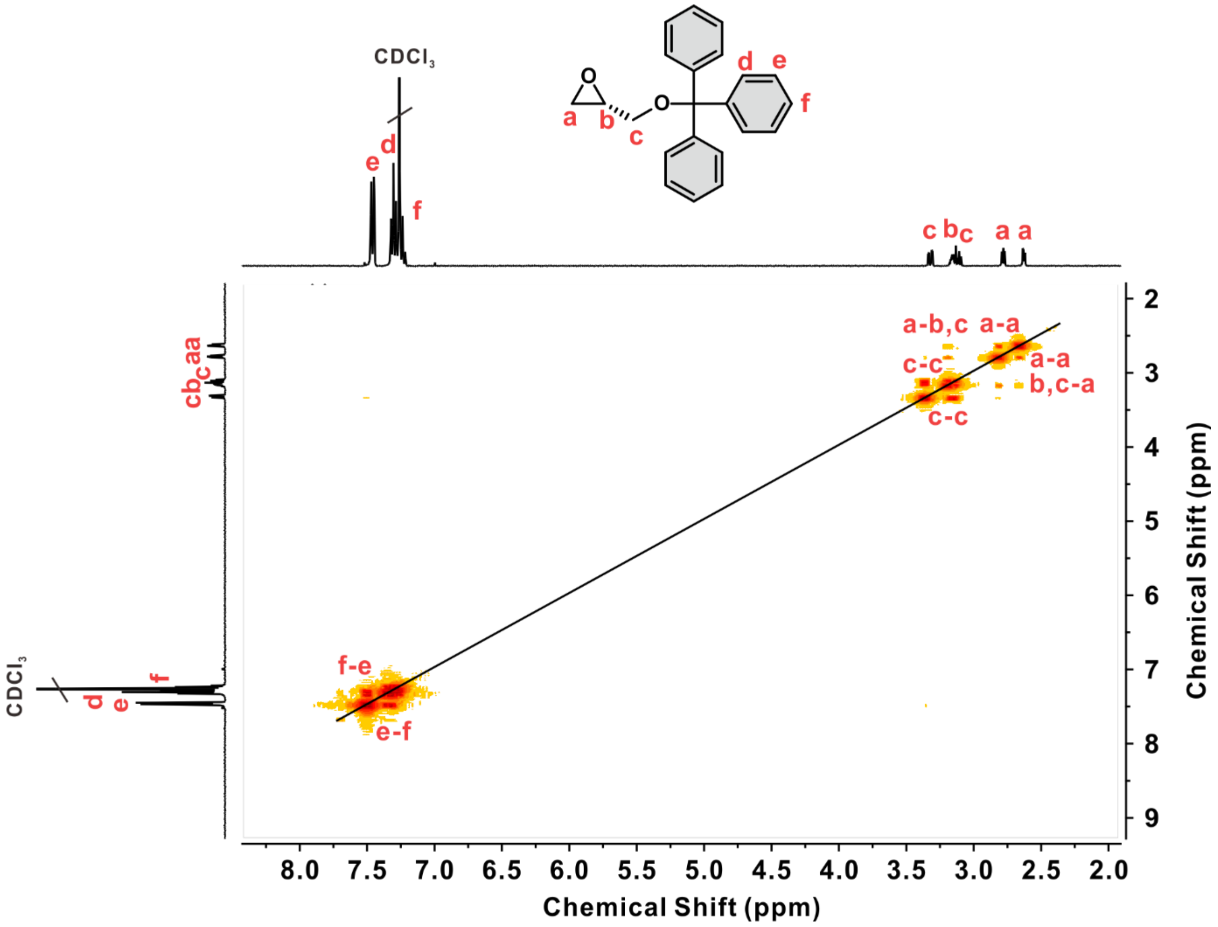

Supplementary Fig. 20. ^1H–^1H COSY NMR spectrum of (*s*)-TGE in $CDCl_3$.

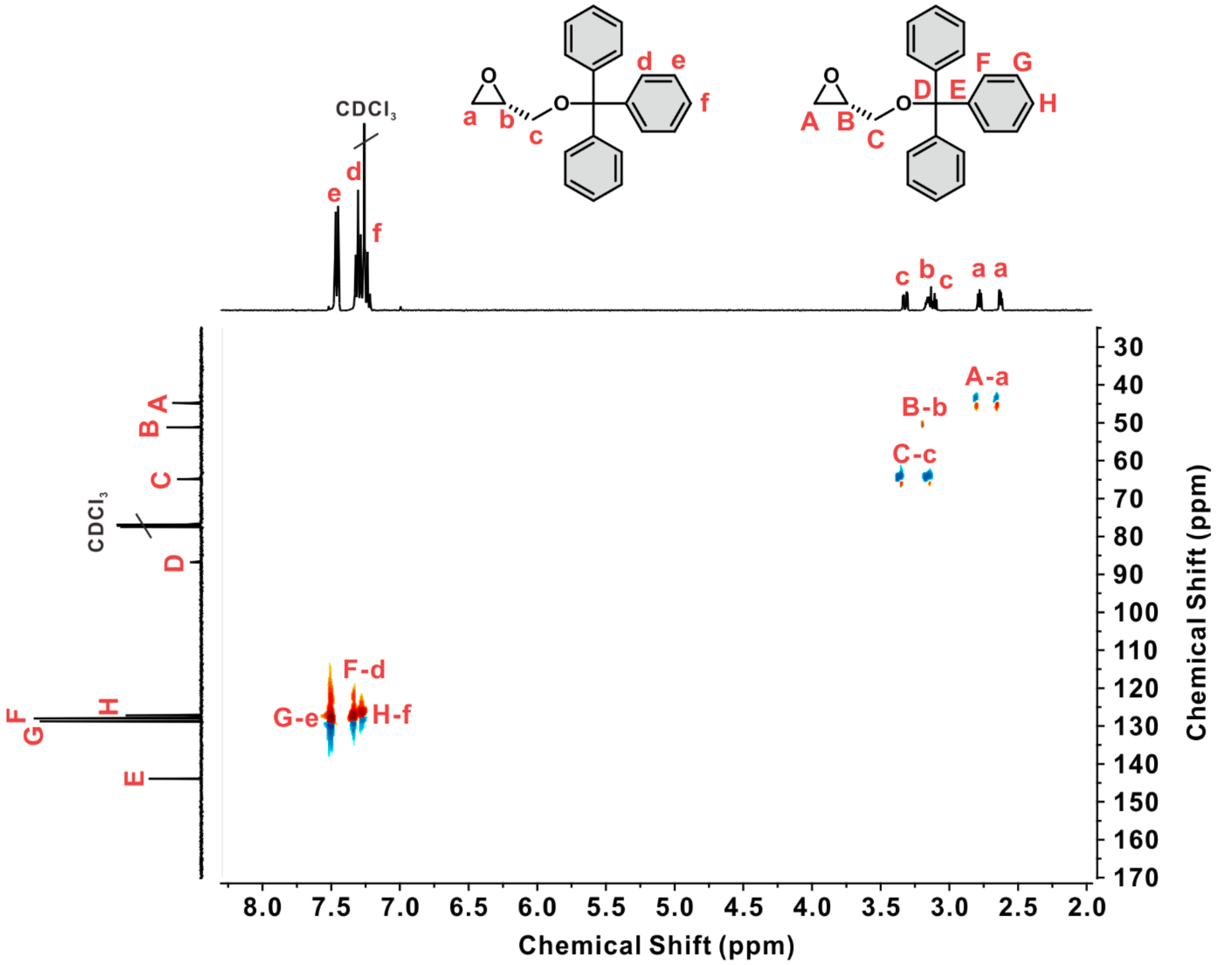

Supplementary Fig. 21. ^{1}H–^{13}C HSQC NMR spectrum of (*s*)-TGE in $CDCl_3$.

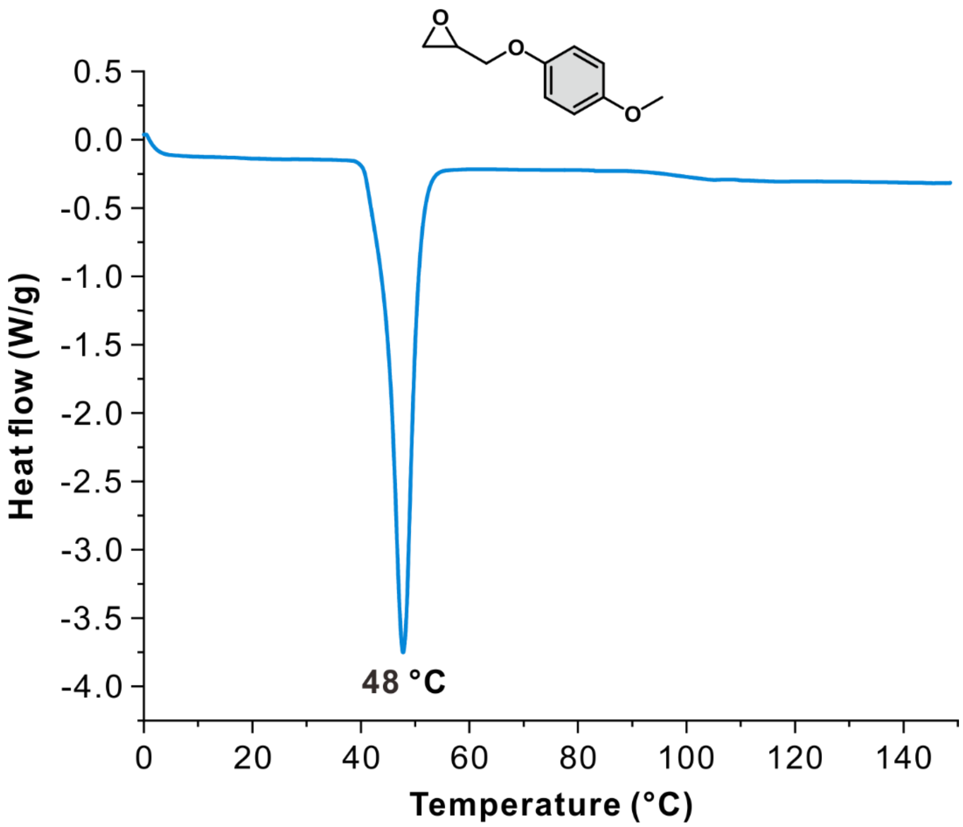

Supplementary Fig. 22. A DSC thermogram of MPG.

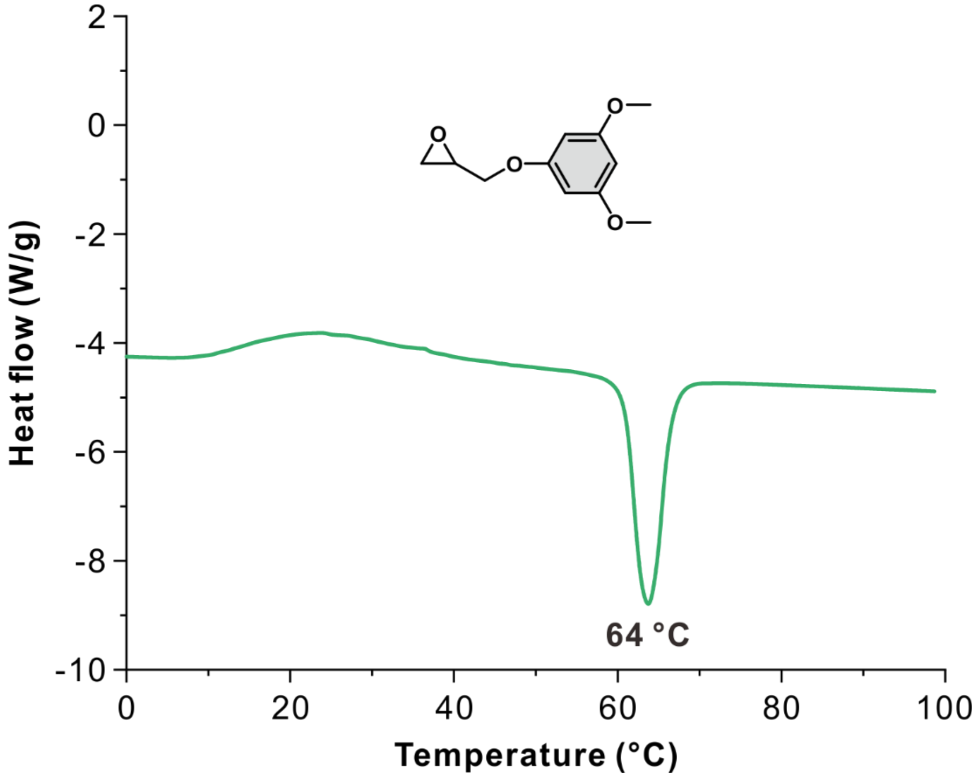

Supplementary Fig. 23. A DSC thermogram of DPG.

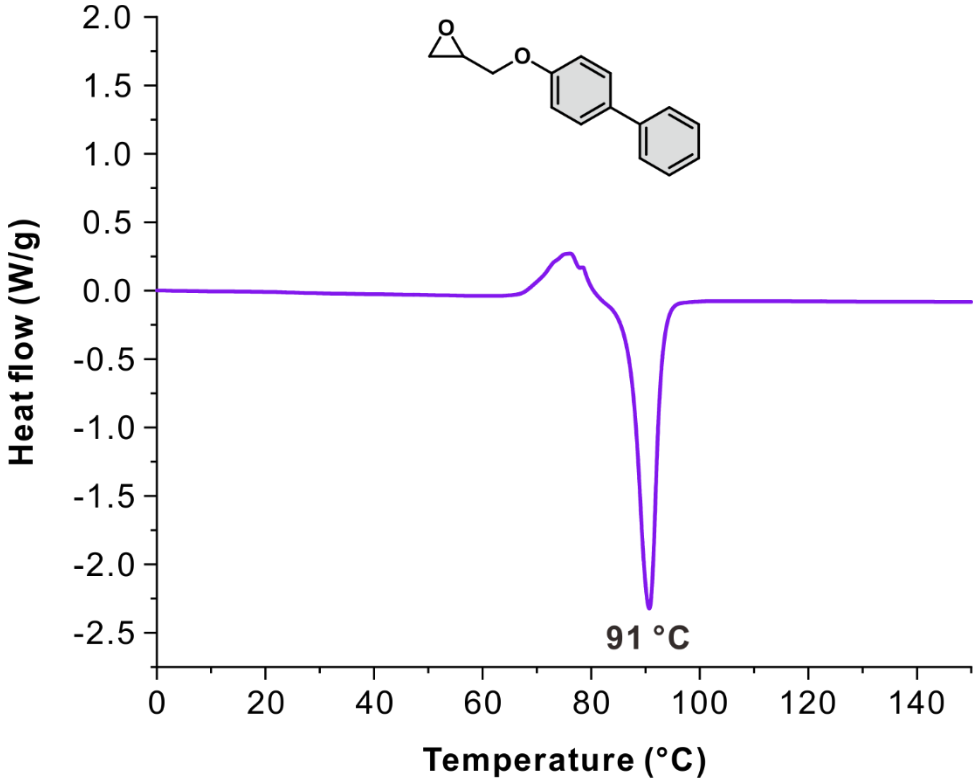

Supplementary Fig. 24. A DSC thermogram of BPG.

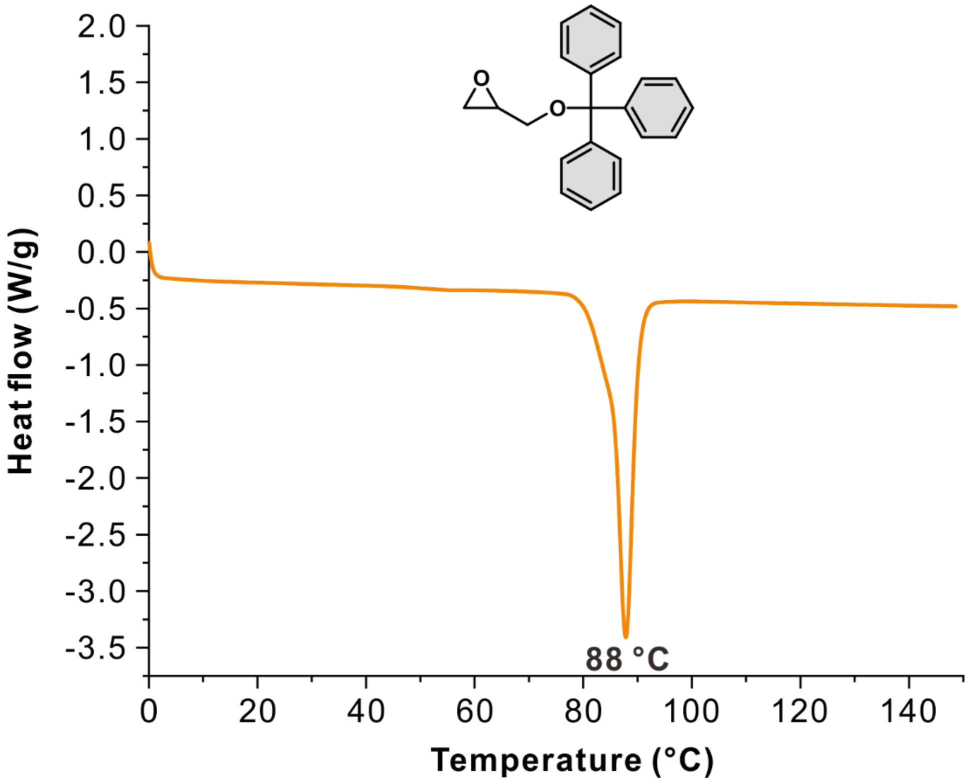

Supplementary Fig. 25. A DSC thermogram of TGE.

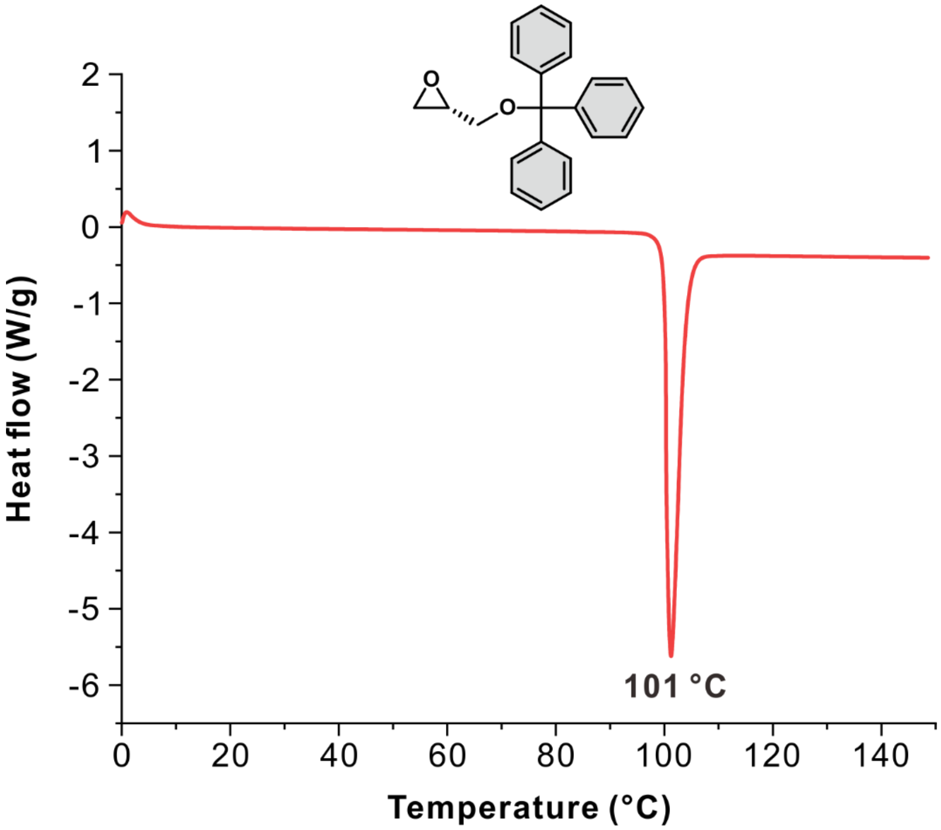

Supplementary Fig. 26. A DSC thermogram of (*s*)-TGE.

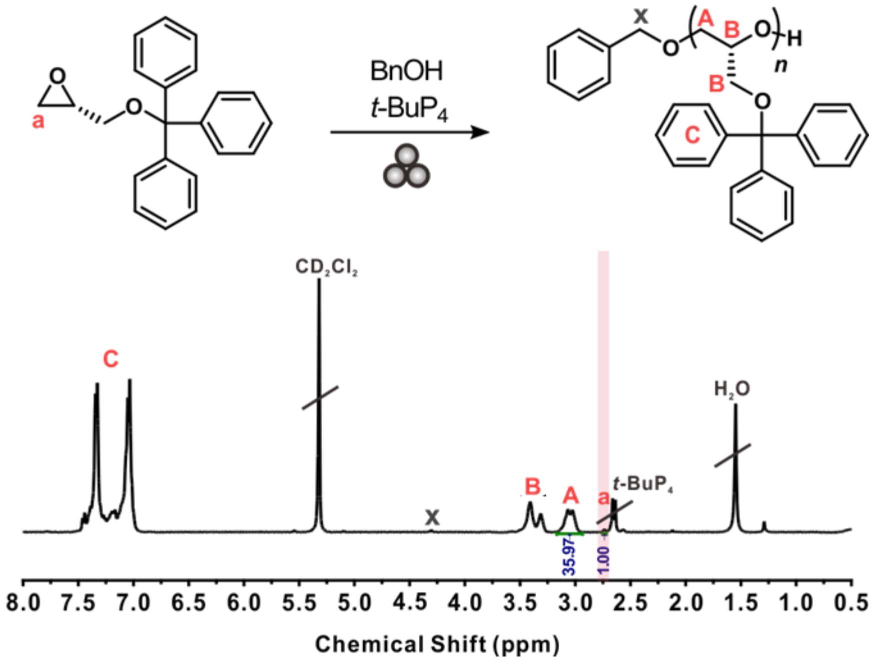

Supplementary Fig. 27. ^{1}H NMR spectrum of (*s*)-PTGE polymer obtained via ball milling AROP for 2 h (400 MHz, CD_2Cl_2): Conv. = 94.4%.

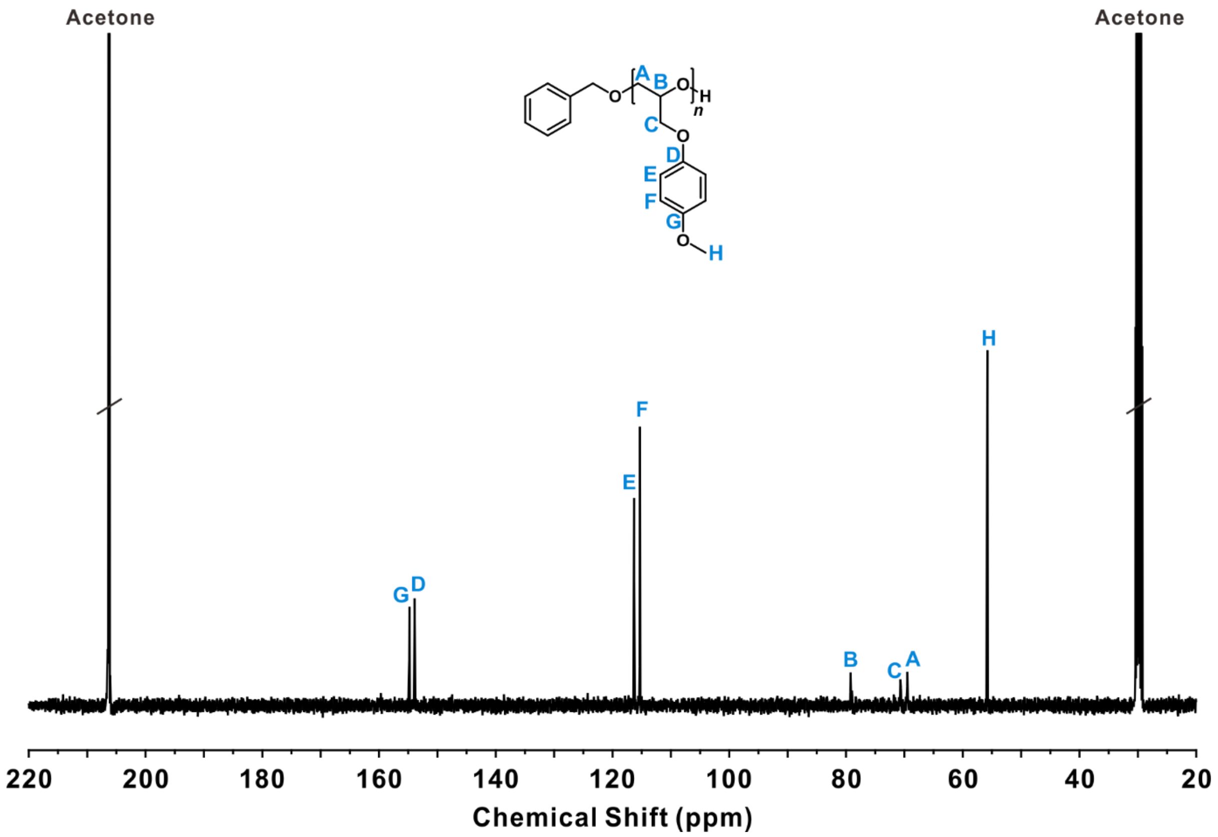

Supplementary Fig. 28. ^{13}C NMR spectrum of PMPG (101 MHz, acetone-d_6).

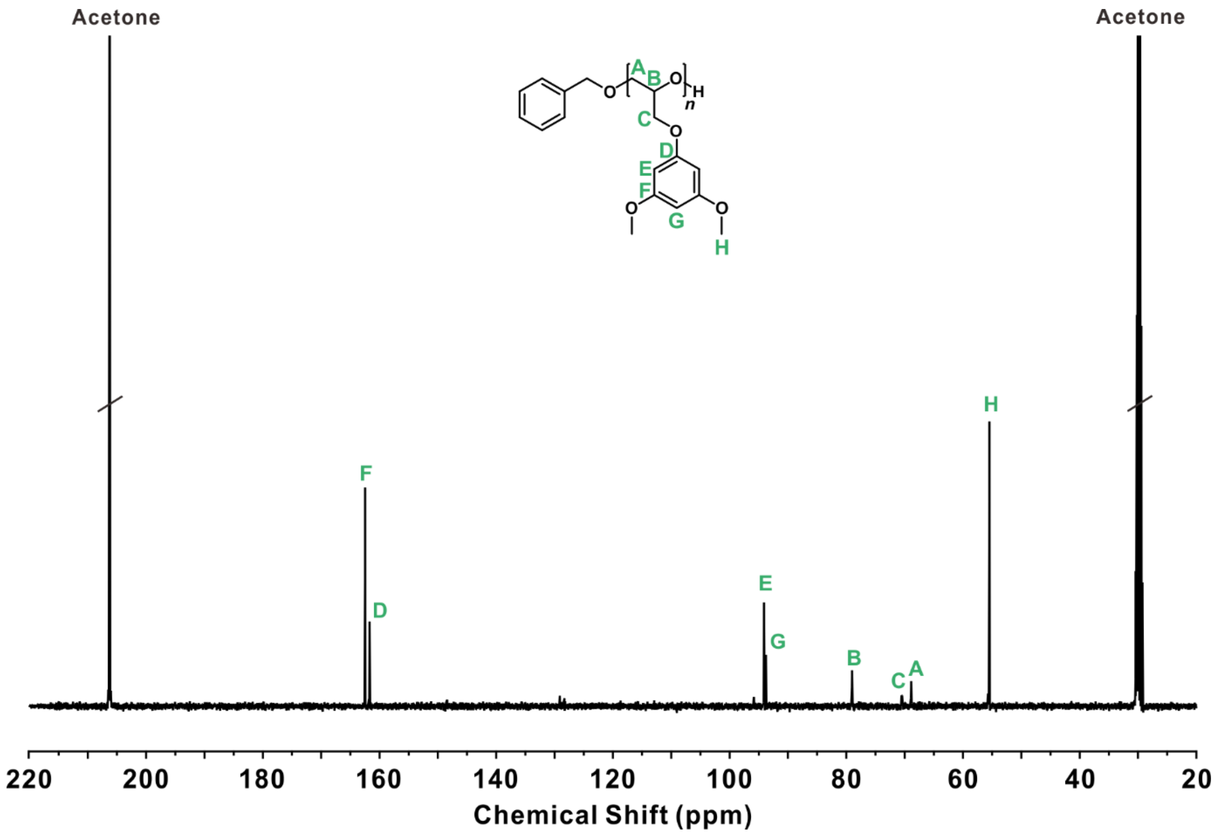

Supplementary Fig. 29. ^{13}C NMR spectrum of PDPG (101 MHz, acetone-d_6).

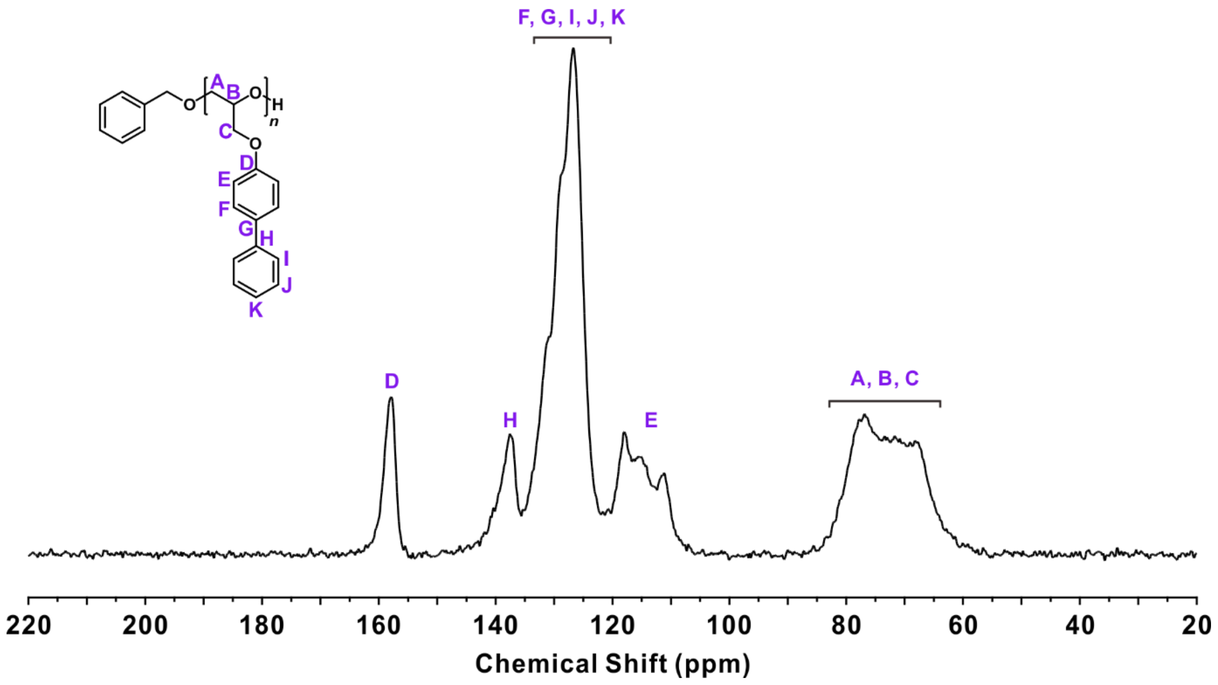

Supplementary Fig. 30. Solid-state ^{13}C NMR spectrum of PBPG.

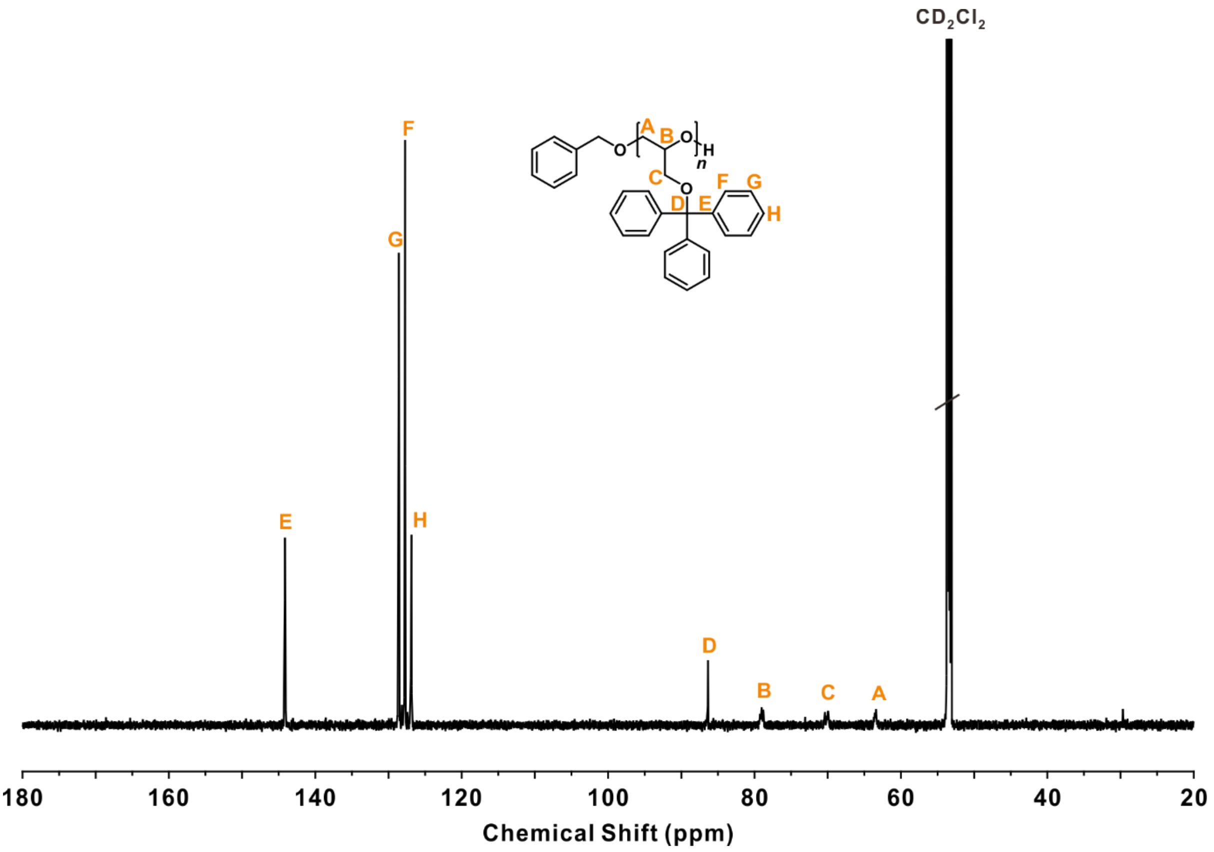

Supplementary Fig. 31. ^{13}C NMR spectrum of PTGE (201 MHz, CD_2Cl_2).

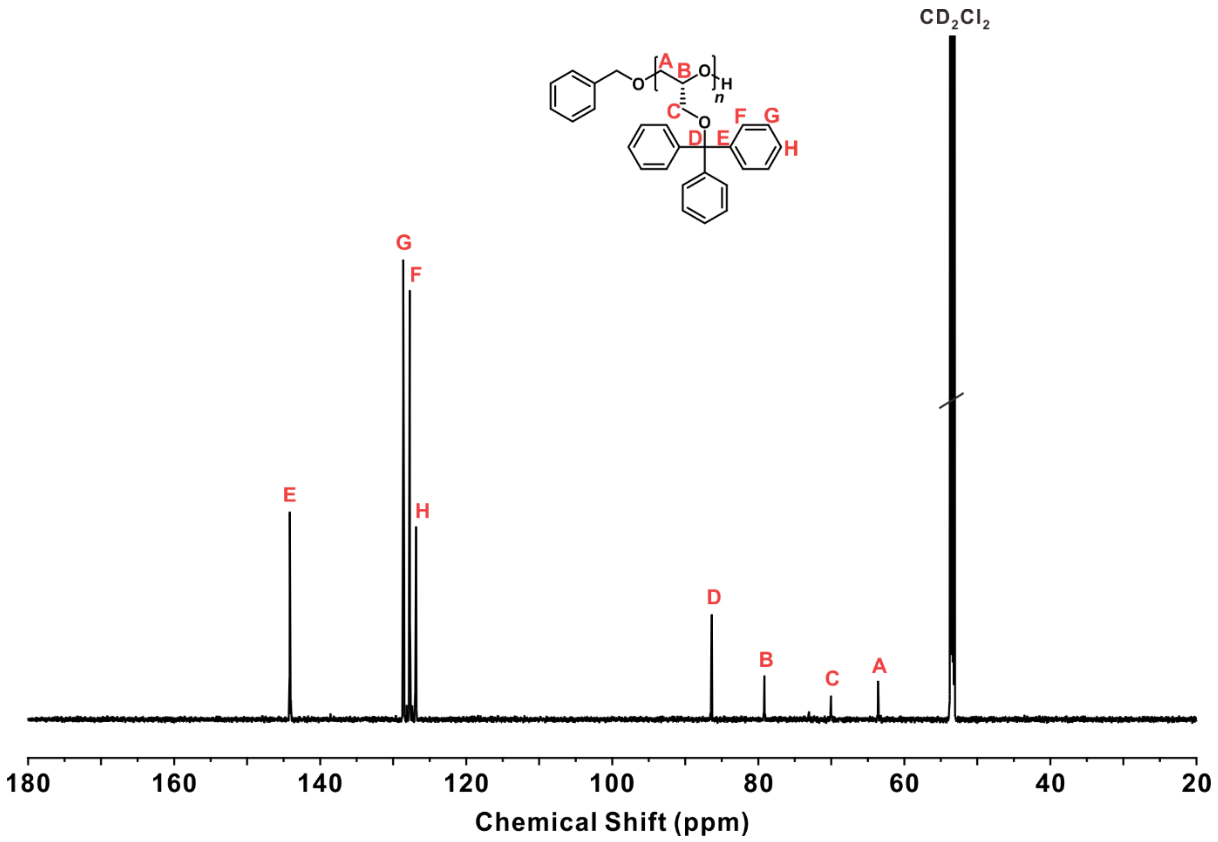

Supplementary Fig. 32. ^{13}C NMR spectrum of (*s*)-PTGE (201 MHz, CD_2Cl_2).

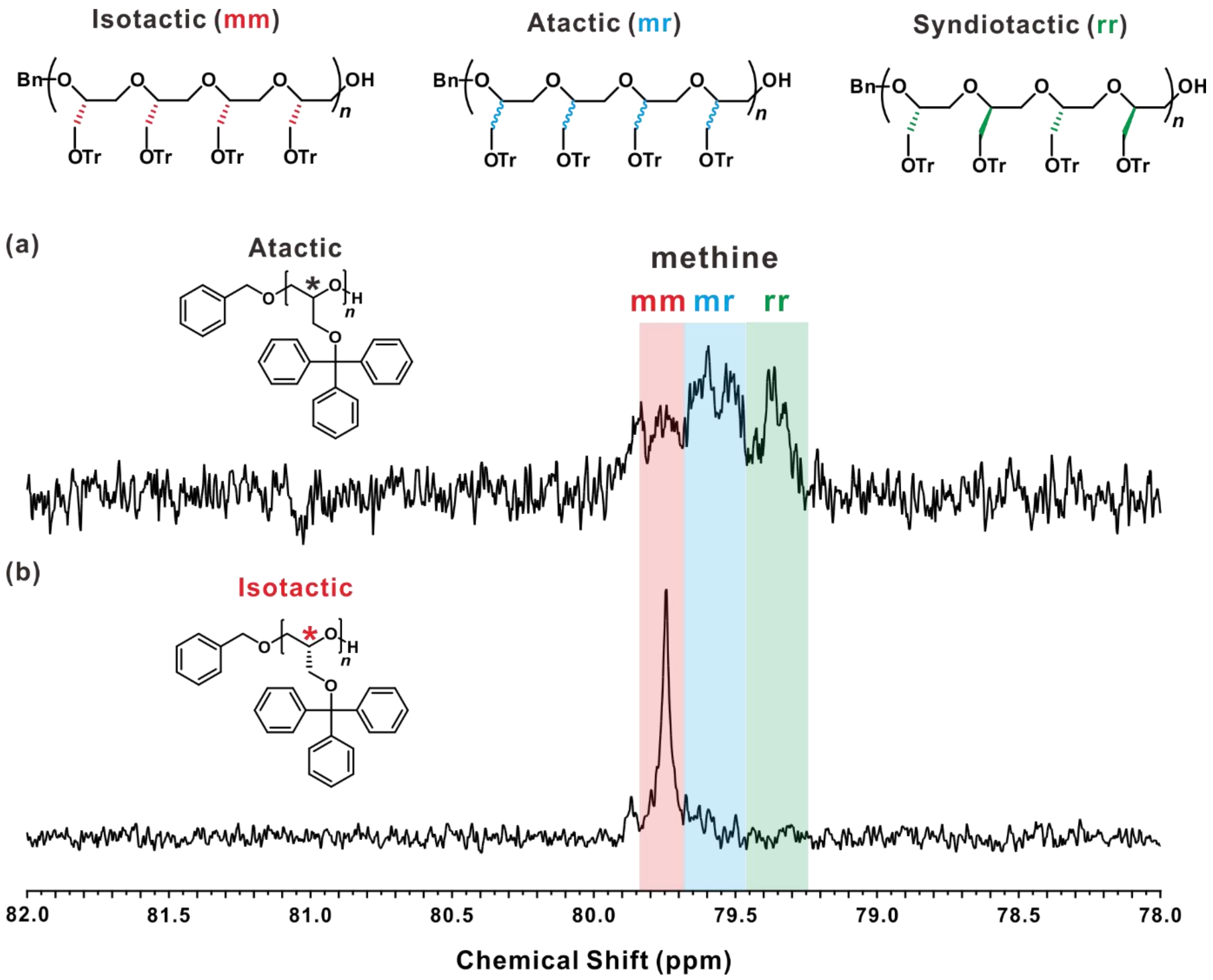

Supplementary Fig. 33. Assignment of methine peaks in the ^{13}C NMR spectra of (a) atactic PTGE and (b) isotactic (*s*)-PTGE (201 MHz, CD_2Cl_2).

Supplementary Table 1. Investigation of ball milling parameters. (a) Effect of type of jar and balls, (b) effect of size of balls, and (c) effect of the number of balls. All polymerization was performed using MPG monomer.

(a)

Entry	Type of jar & balls	Size of balls (mm)	# of balls	Time (h)	Conv. (%)	DP_{NMR}
**1**	Teflon & ZrO_2	5	4	6	33	8
**2**	Stainless & Stainless	7	3	6	>99	100

(b)

Entry	Type of jar & balls	Size of balls (mm)	# of balls	Time (h)	Conv. (%)	DP_{NMR}
**1**	Stainless & Stainless	7	3	0.5	20	n.d.
**2**	Stainless & Stainless	12	3	0.5	48	176

(c)

Entry	Type of jar & balls	Size of balls (mm)	# of balls	Time (h)	Conv. (%)	DP_{NMR}
**1**	Stainless & Stainless	7	3	6	>99	100
**2**	Stainless & Stainless	7	10	4	>99	130
**3**	Stainless & Stainless	7	20	2	>99	366

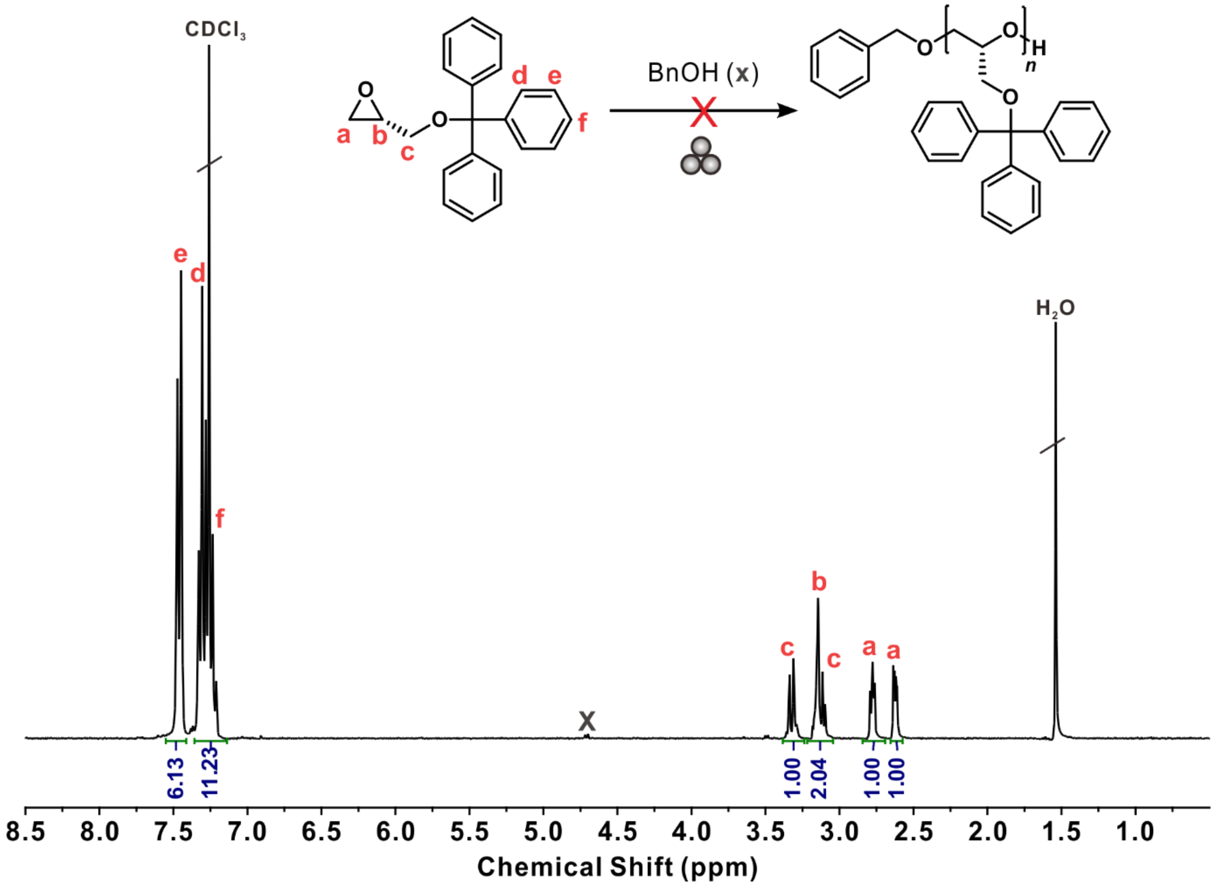

Supplementary Fig. 34. ^{1}H NMR spectrum of ball milling AROP of (*s*)-PTGE$_{15}$ in the absence of *t*-BuP$_4$ base (400 MHz, $CDCl_3$).

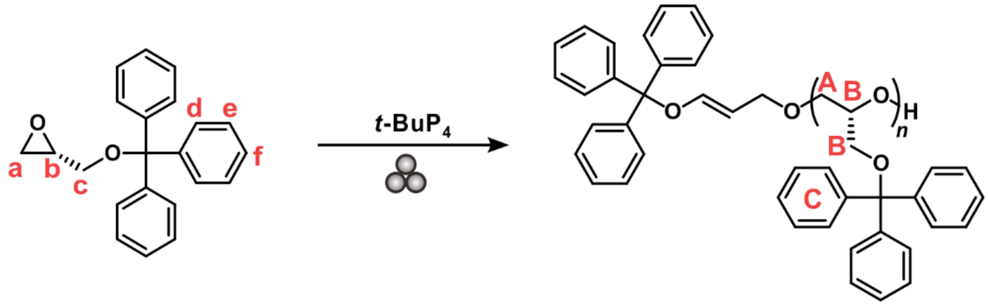

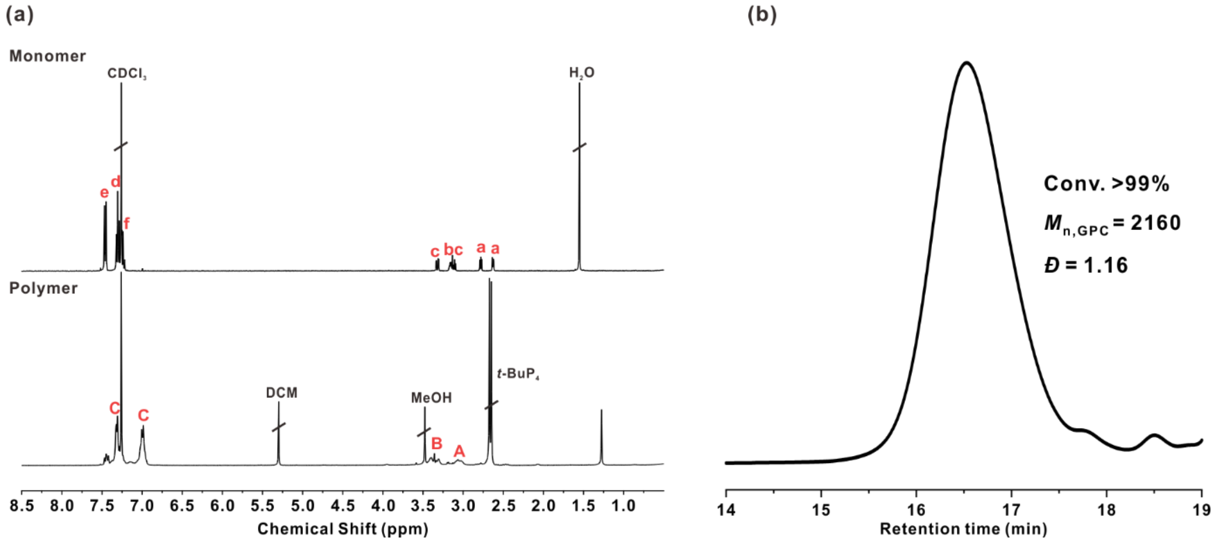

Supplementary Fig. 35. Ball milling AROP of (*s*)-PTGE$_{15}$ without benzyl alcohol initiator. (a) ^{1}H NMR spectrum (400 MHz, $CDCl_3$), (b) GPC trace measured in THF using RI signal with PS standards.

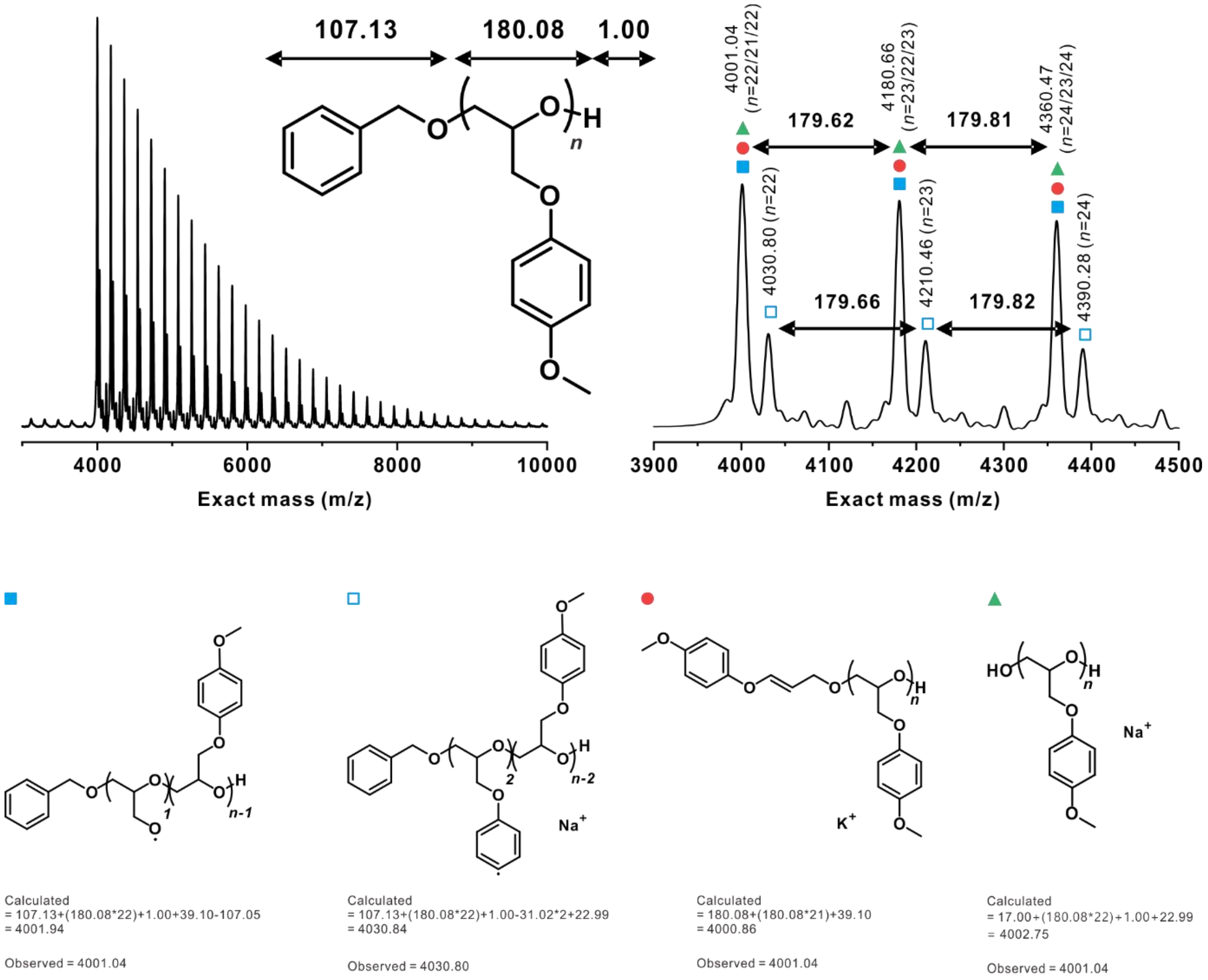

Supplementary Fig. 36. MALDI-ToF MS spectrum of the isolated PMPG polyether with individual peak assignments in the selected region. Experimental conditions: linear positive mode, and 2,5-dihydroxybenzoic acid matrix.

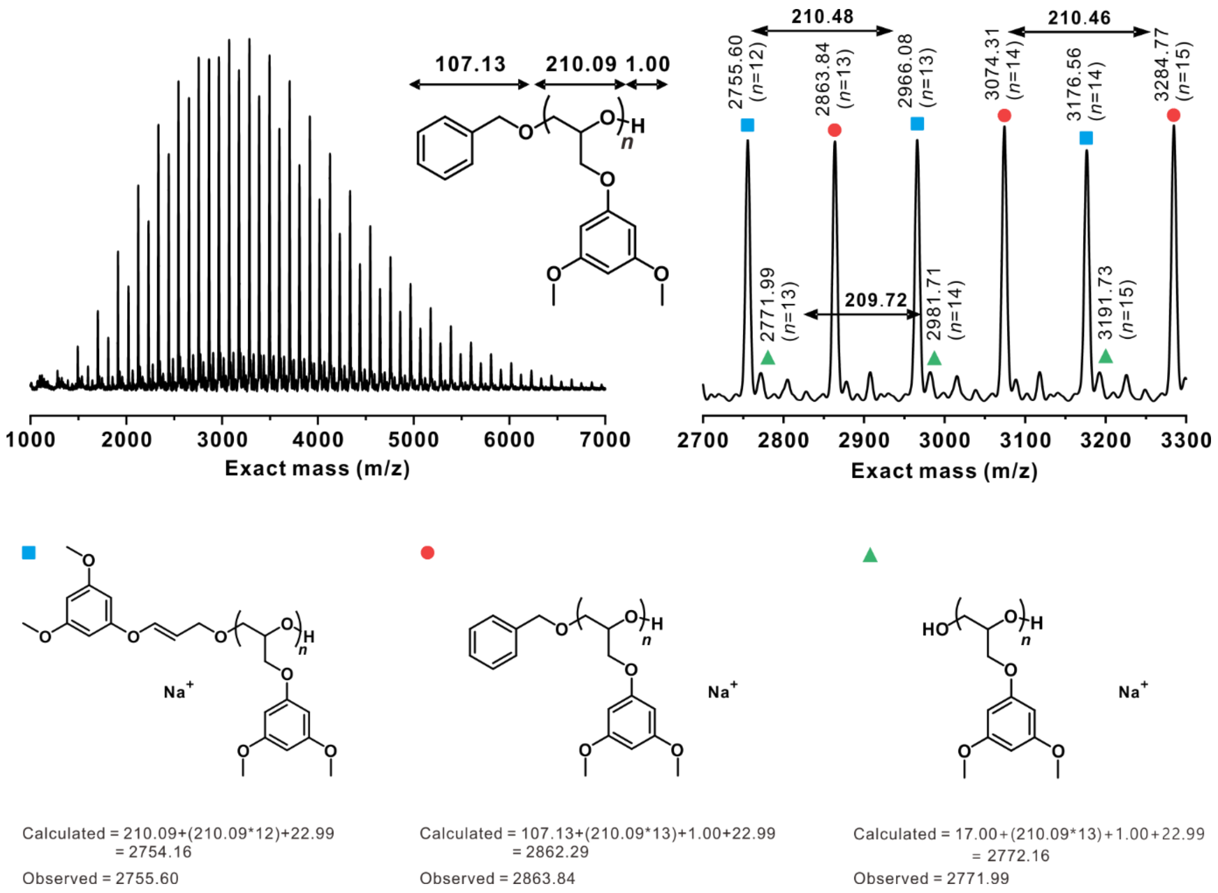

Supplementary Fig. 37. MALDI-ToF MS spectrum of the isolated PDPG polyether with individual peak assignments in the selected region. Experimental conditions: linear positive mode, and 2,5-dihydroxybenzoic acid matrix.

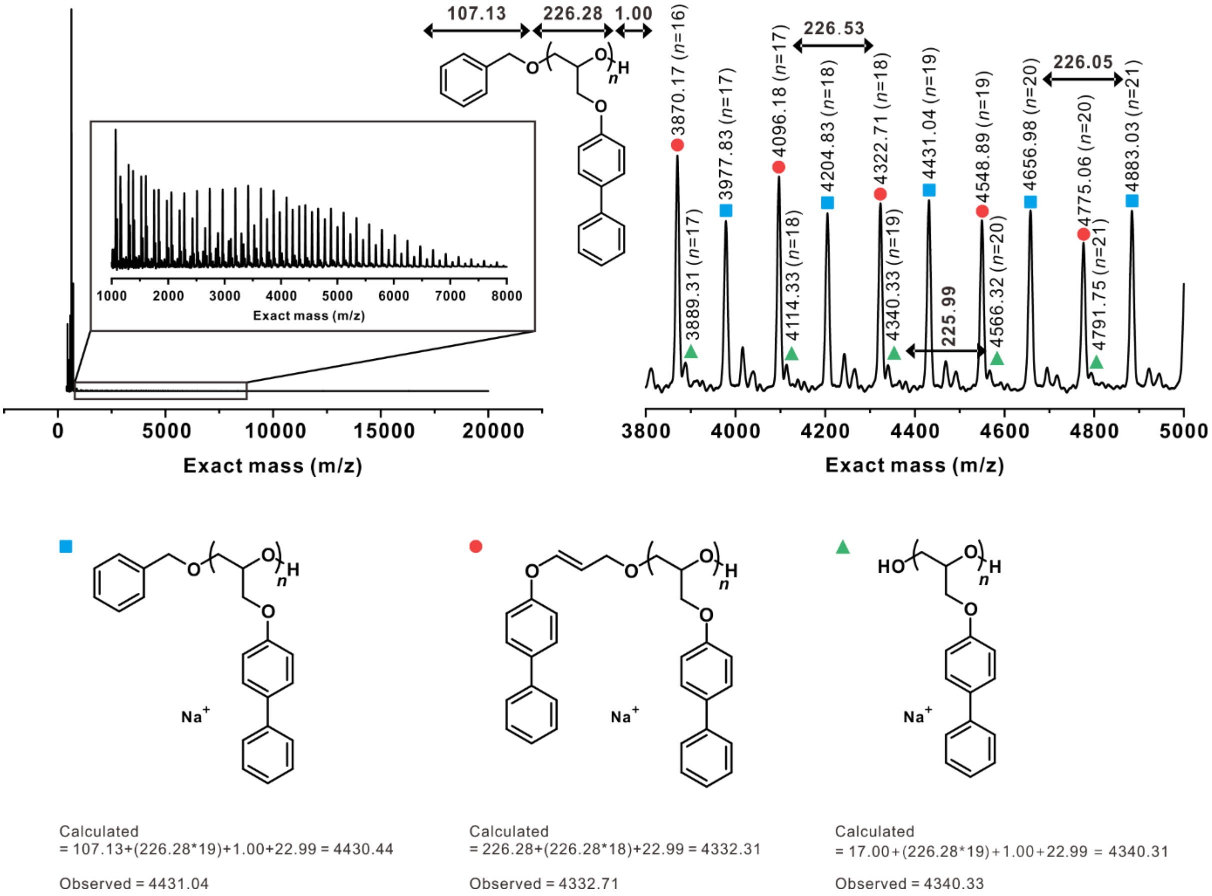

Supplementary Fig. 38. MALDI-ToF MS spectrum of the isolated PBPG polyether with individual peak assignments in the selected region. Experimental conditions: linear positive mode, and 2,5-dihydroxybenzoic acid matrix.

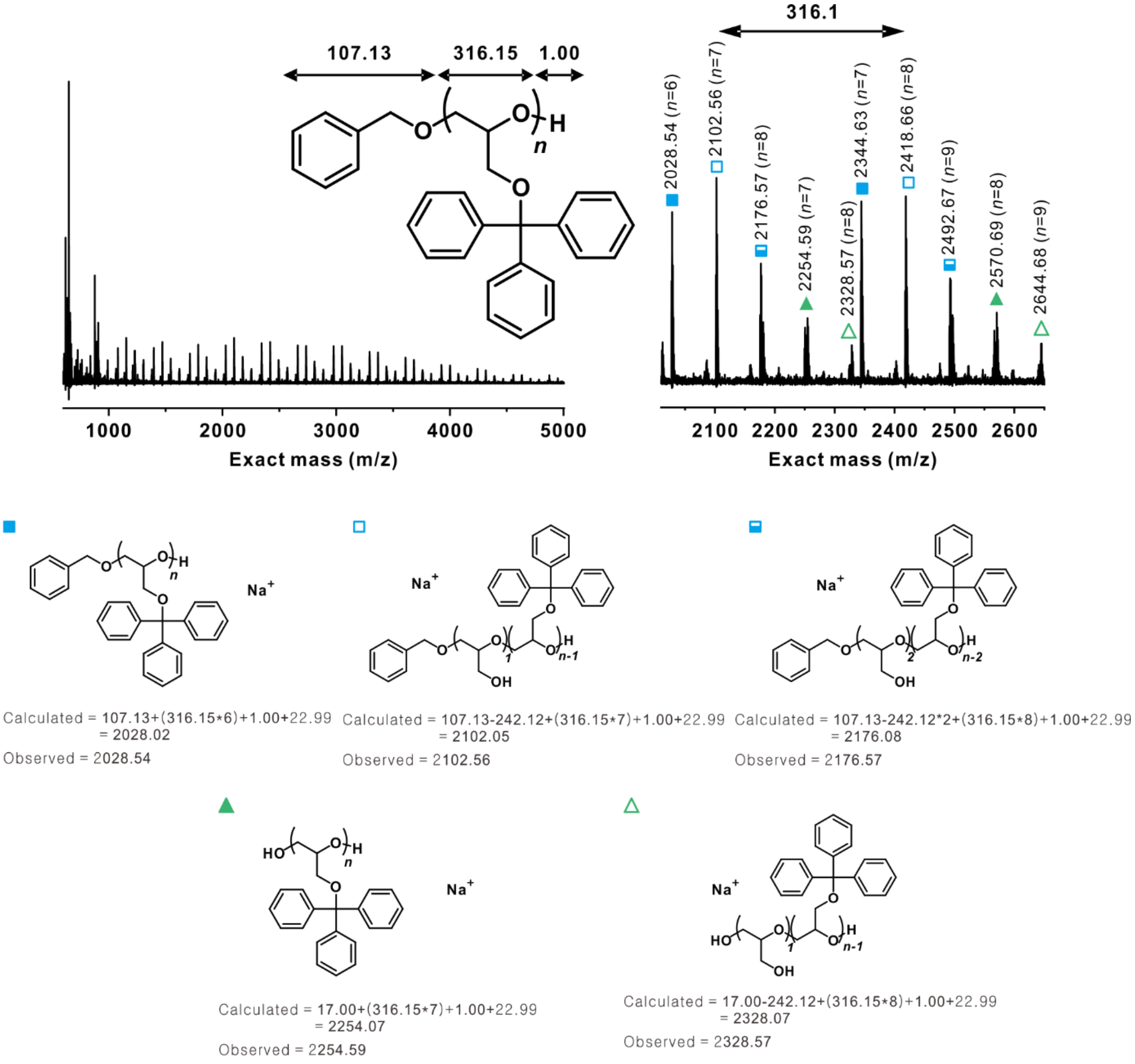

Supplementary Fig. 39. MALDI-ToF MS spectrum of the isolated PTGE polyether with individual peak assignments in the selected region. Experimental conditions: reflector positive mode, and 2,5-dihydroxybenzoic acid matrix.

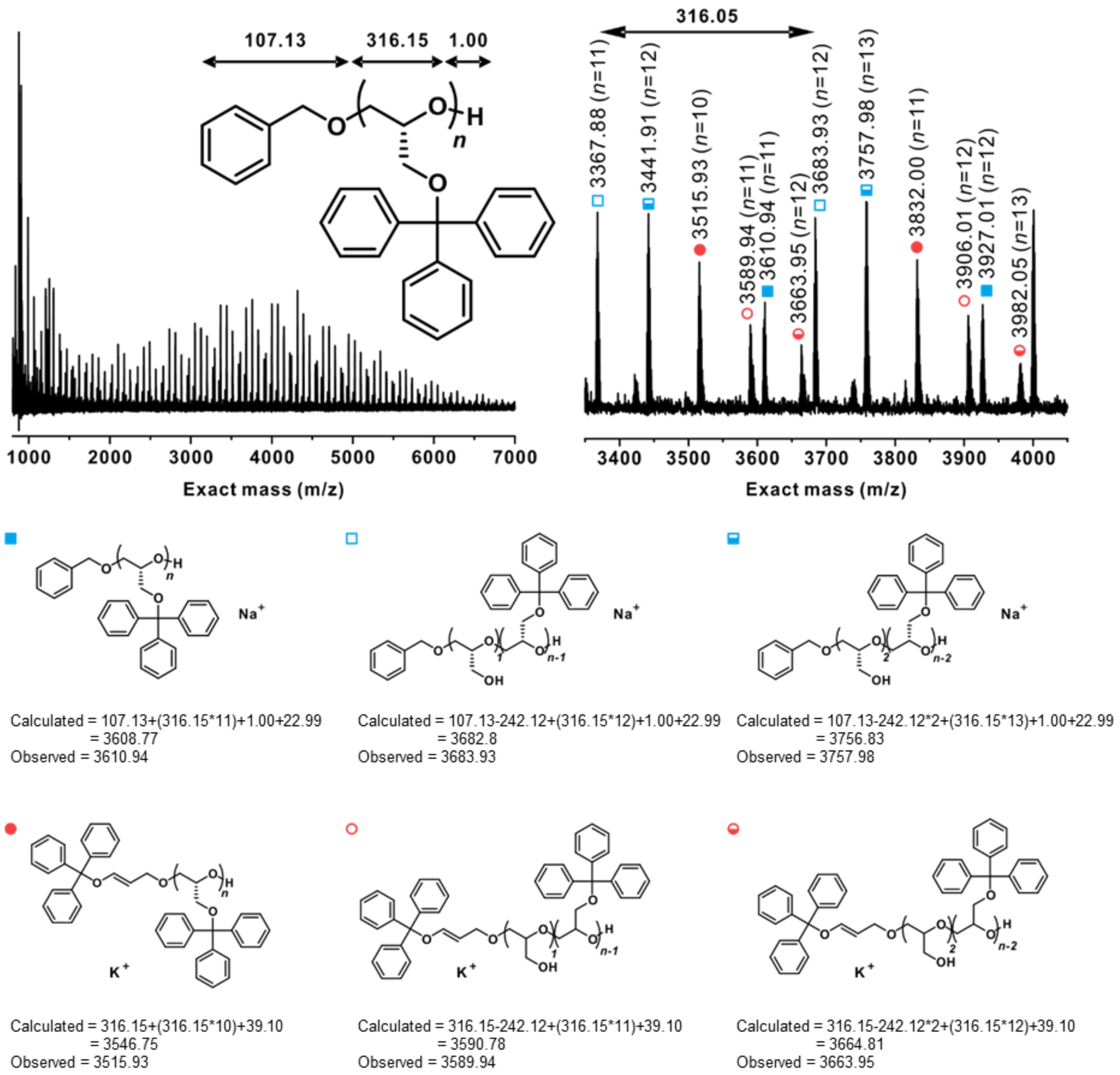

Supplementary Fig. 40. MALDI-ToF MS spectrum of the isolated (*s*)-PTGE polyether with individual peak assignments in the selected region. Experimental conditions: reflector positive mode, and 2,5-dihydroxybenzoic acid matrix.

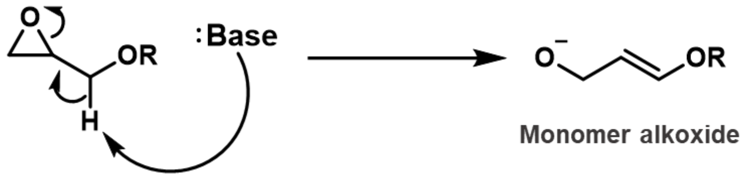

Supplementary Fig. 41. Initiation mechanism for the elimination of monomers.

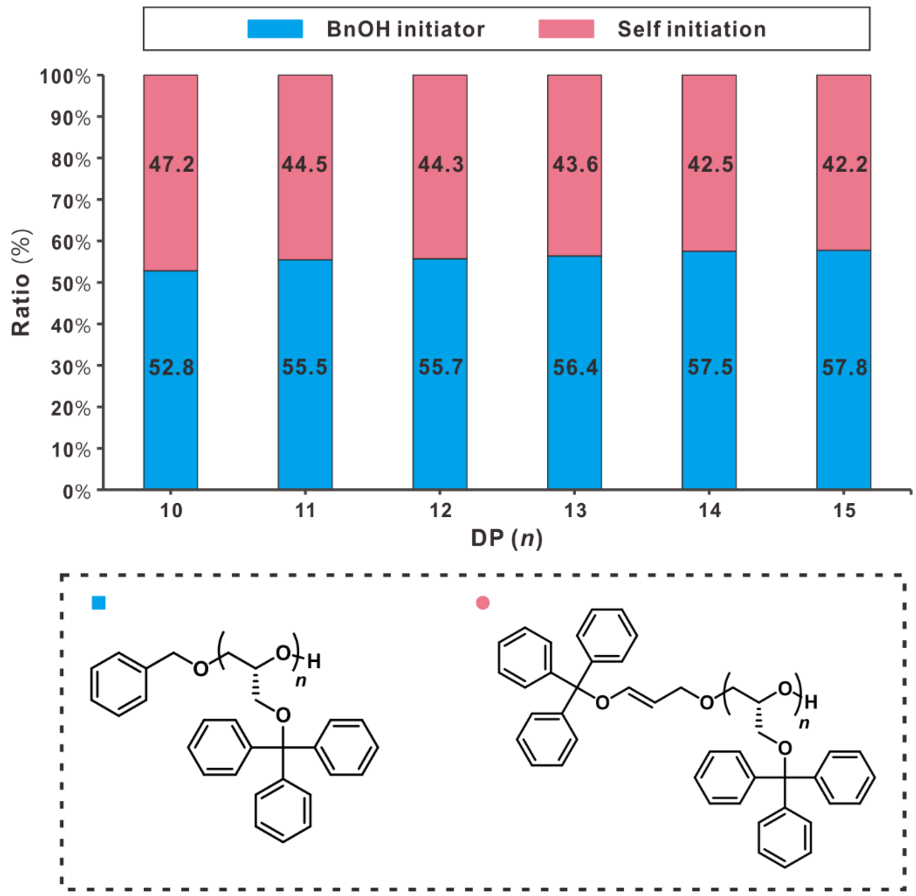

Supplementary Fig. 42. Distribution histogram of individual initiating group in the resulting (*s*)-PTGE polyether displayed in Supplementary Fig. 40.

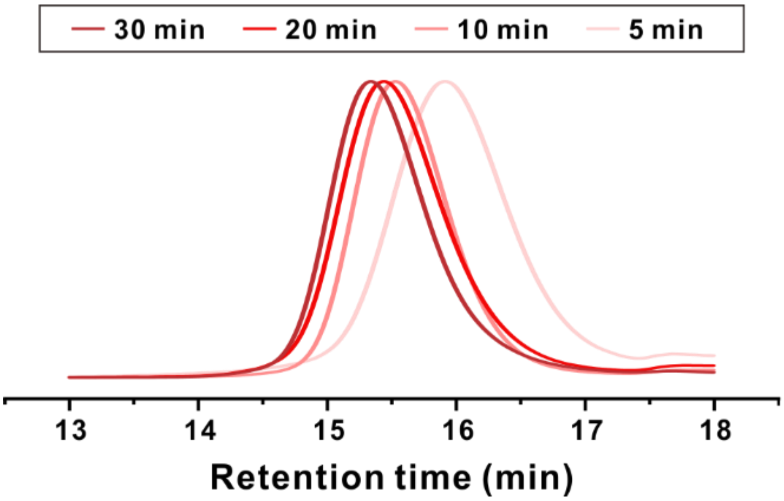

Supplementary Fig. 43. GPC curves of (*s*)-PTGE with different polymerization time of 5, 10, 20, and 30 min measured in THF using RI signal with PS standards.

Supplementary Table 2. Characterization of the synthesized (*s*)-PTGE polymer with different polymerization time.

Polymer	Time (min)	Conv.[a] (%)	M_{GPC}[b] (g/mol)	$Đ$[b]
(*s*)-PTGE	5	46.9	3820	1.15
	10	78.9	5400	1.10
	20	92.9	5600	1.14
	30	92.1	5950	1.15

[a]Monomer conversion as calculated from the ^{1}H NMR spectrum of the crude monomer. [b]Measured via GPC in THF using PS standard.

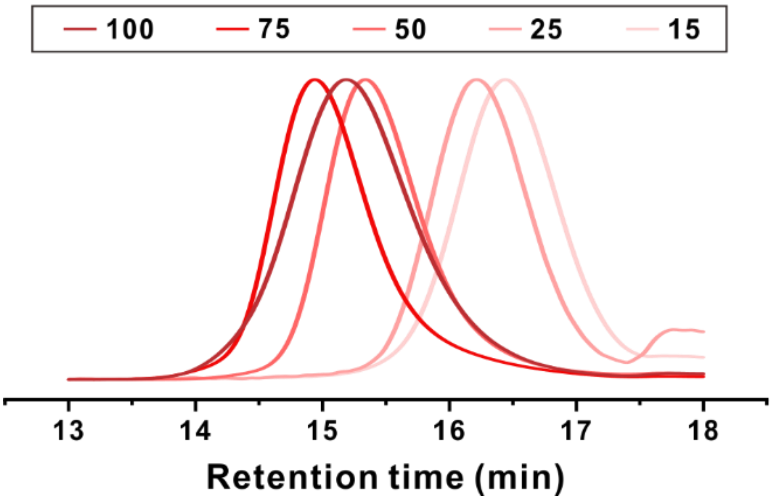

Supplementary Fig. 44. GPC curves of (*s*)-PTGE with different degree of polymerization of 15, 25, 50, 75, and 100 measured in THF using RI signal with PS standards.

Supplementary Table 3. Characterization of the synthesized (*s*)-PTGE polymer with different degree of polymerization.

Polymer	Time (min)	Target DP	Conv.[a] (%)	M_{GPC}[b] (g/mol)	$Đ$[b]
	30	15	>99	2550	1.11
	30	25	90.7	3100	1.09
(*s*)-PTGE	30	50	92.1	5950	1.14
	30	75	87.1	7530	1.21
	30	100	71.0	6660	1.21

[a]Monomer conversion as calculated from the ^{1}H NMR spectrum of the crude monomer. [b]Measured via GPC in THF using PS standard.

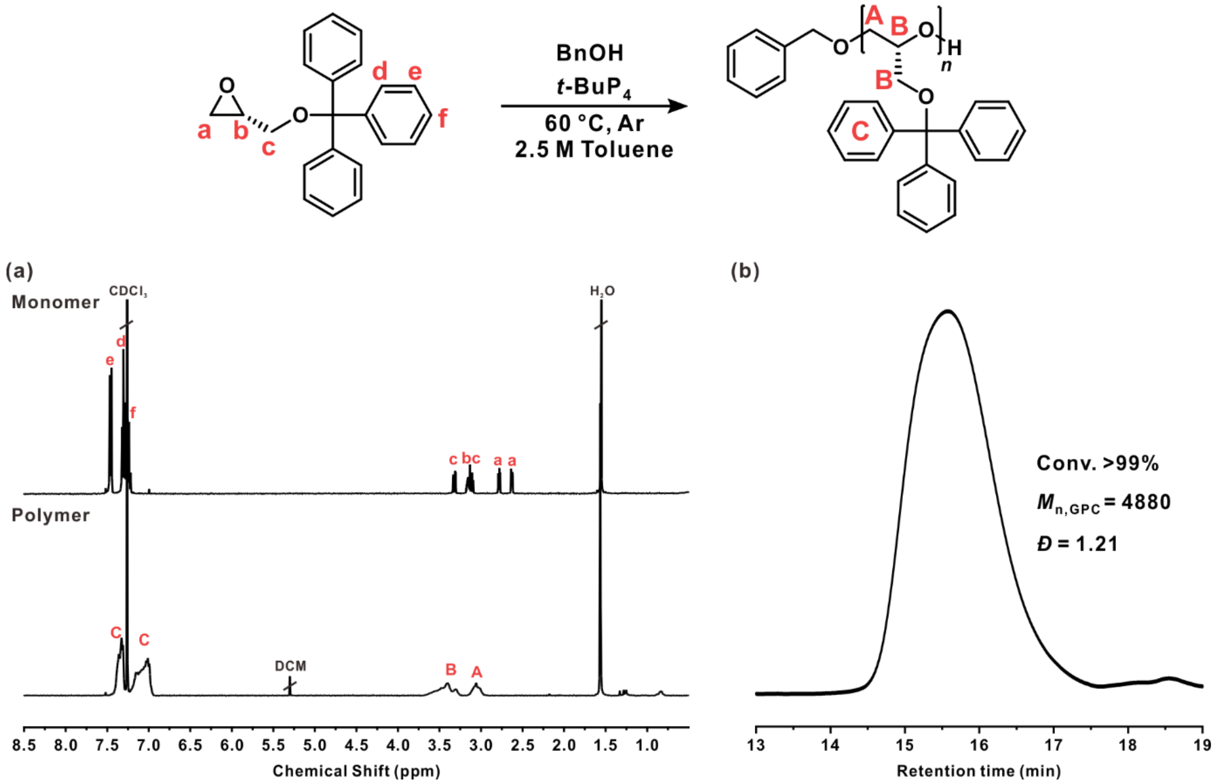

Supplementary Fig. 45. Synthesis of (*s*)-PTGE via the conventional solution polymerization. (a) ^{1}H NMR spectra (400 MHz, $CDCl_3$), and (b) GPC trace of (*s*)-PTGE polymer.

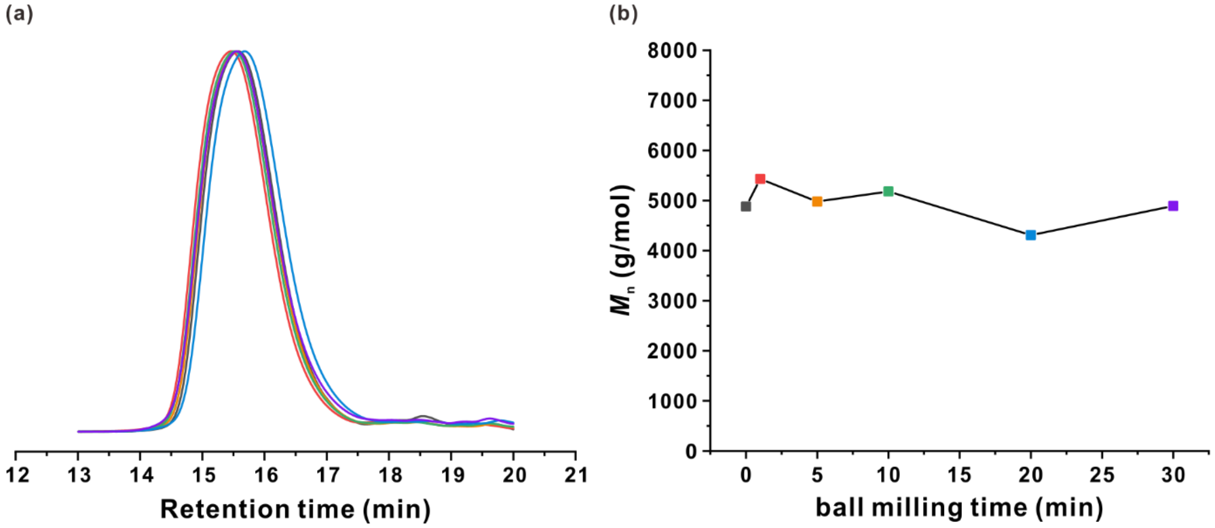

Supplementary Fig. 46. Ball milling degradation of (*s*)-PTGE prepared via solution polymerization. (a) GPC curves of (*s*)-PTGE with different degradation times of 1 min (red), 5 min (orange), 10 min (green), 20 min (blue), and 30 min (purple). (b) Changes in M_n measured by GPC as a function of ball milling time for degradation of (*s*)-PTGE prepared via solution polymerization.

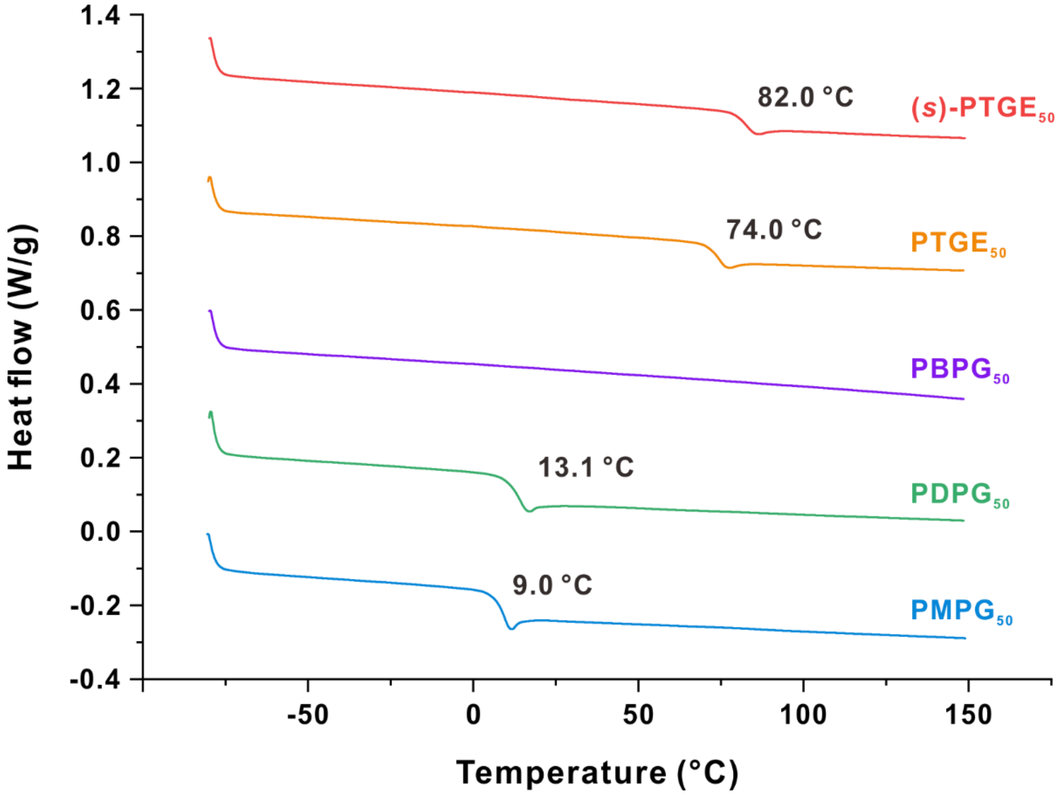

Supplementary Fig. 47. DSC thermograms of the obtained polyethers determined via DSC between –80 and 150 °C at a rate of 10 °C/min. Note that no T_g was observed for $PBPG_{50}$ (see Supplementary Fig. 48).

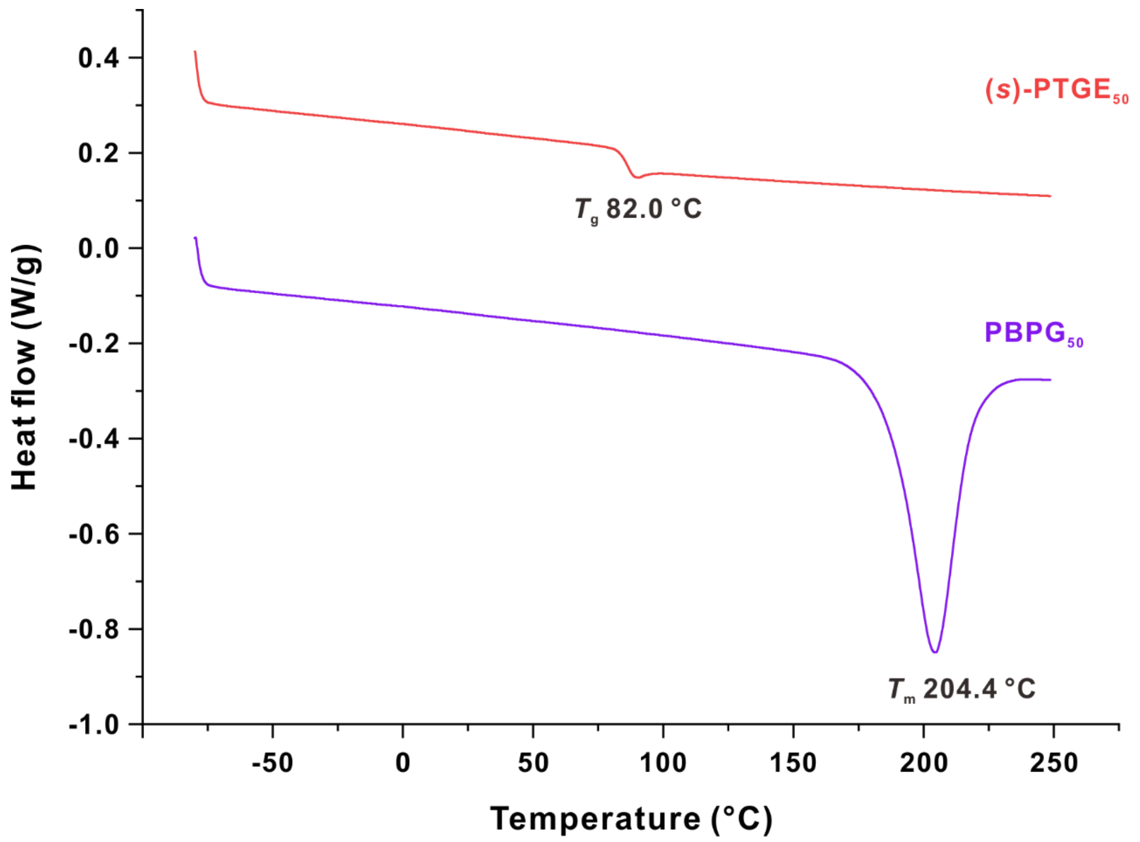

Supplementary Fig. 48. DSC thermograms of the obtained PBPG polymer (purple) and (*s*)-PTGE polymer (red) determined via DSC between –80 and 250 °C at a rate of 10 °C/min.

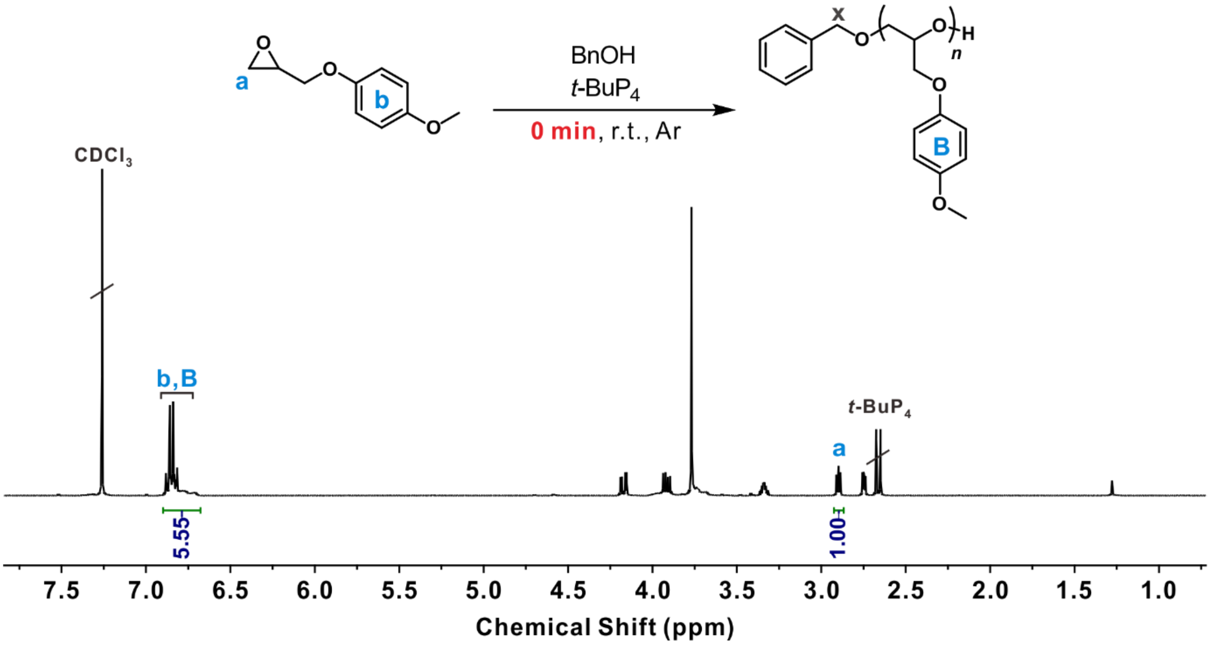

Supplementary Fig. 49. ^{1}H NMR spectrum of initial bulk polymerization of MPG monomer (400 MHz, $CDCl_3$): Conv. = 27.9%. Initial bulk polymerization occurred in the preparation of stainless jar containing reactants and three stainless-steel balls in glove box before performing ball milling reaction.

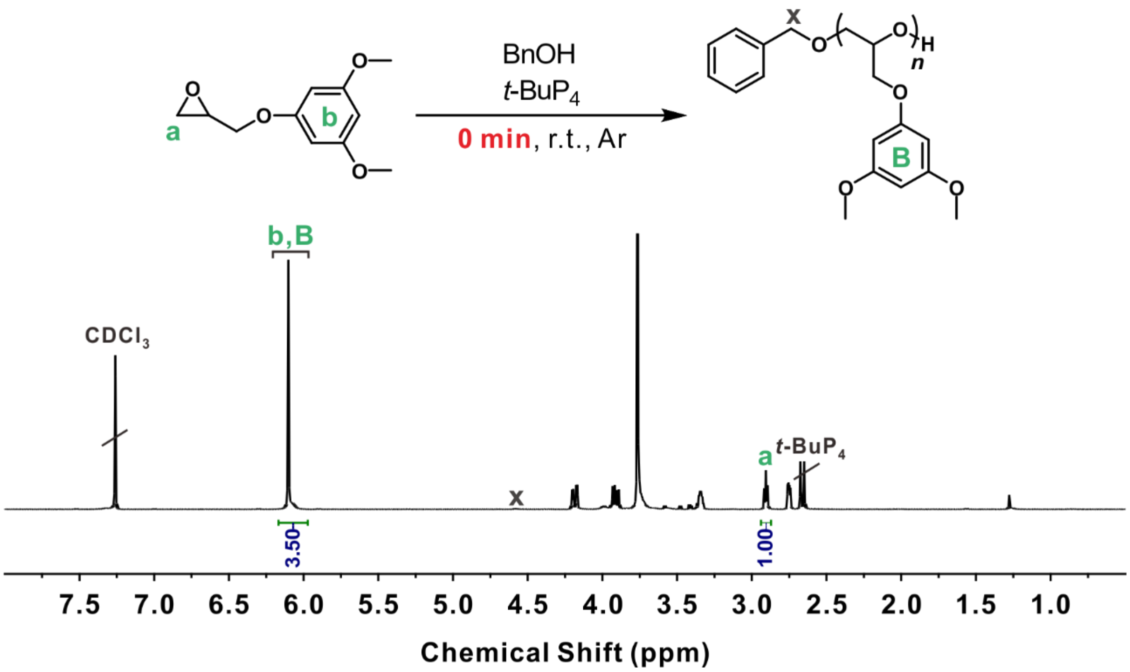

Supplementary Fig. 50. ^{1}H NMR spectrum of initial bulk polymerization of DPG monomer (400 MHz, $CDCl_3$): Conv. = 14.3%. Initial bulk polymerization occurred in the preparation of stainless jar containing reactants and three stainless-steel balls in glove box before performing ball milling reaction.

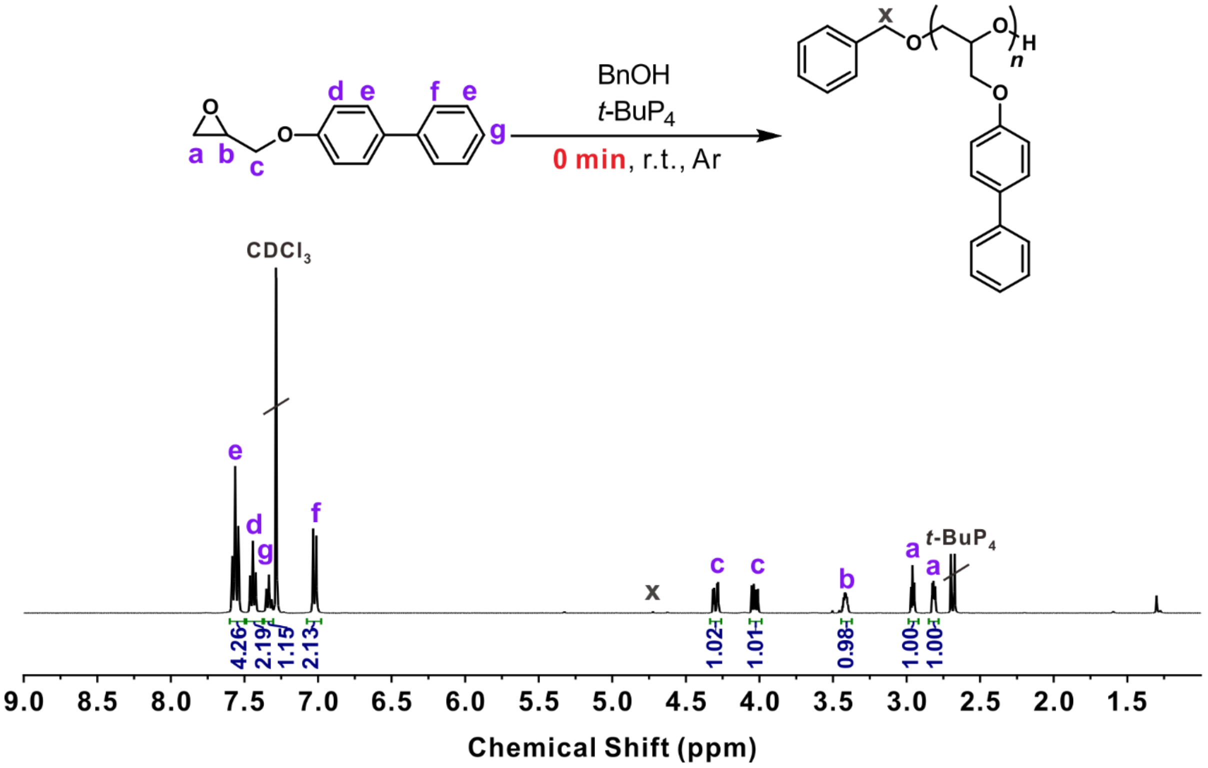

Supplementary Fig. 51. ^{1}H NMR spectrum of initial bulk polymerization of BPG monomer (400 MHz, $CDCl_3$): Conv. = 0.0%. Initial bulk polymerization occurred in the preparation of stainless jar containing reactants and three stainless-steel balls in glove box before performing ball milling reaction.

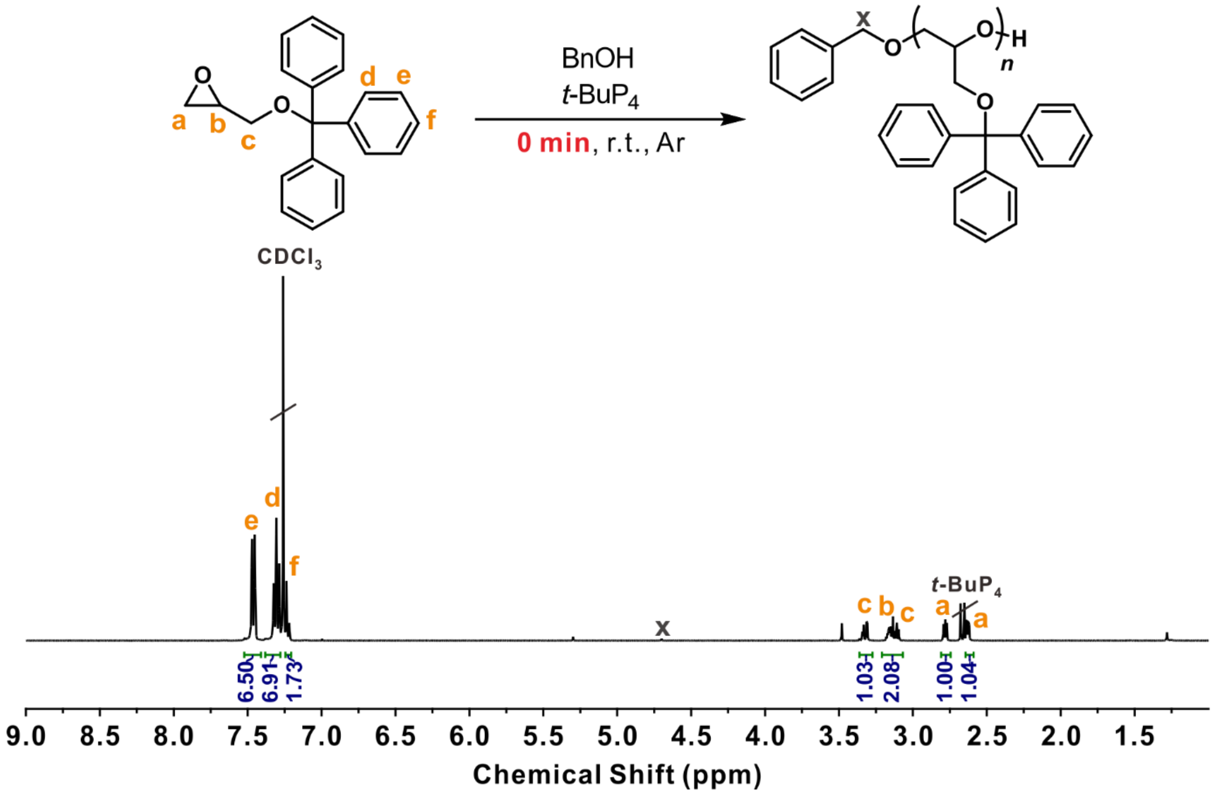

Supplementary Fig. 52. ^{1}H NMR spectrum of initial bulk polymerization of TGE monomer (400 MHz, $CDCl_3$): Conv. = 0.0%. Initial bulk polymerization occurred in the preparation of stainless jar containing reactants and three stainless-steel balls in glove box before performing ball milling reaction.

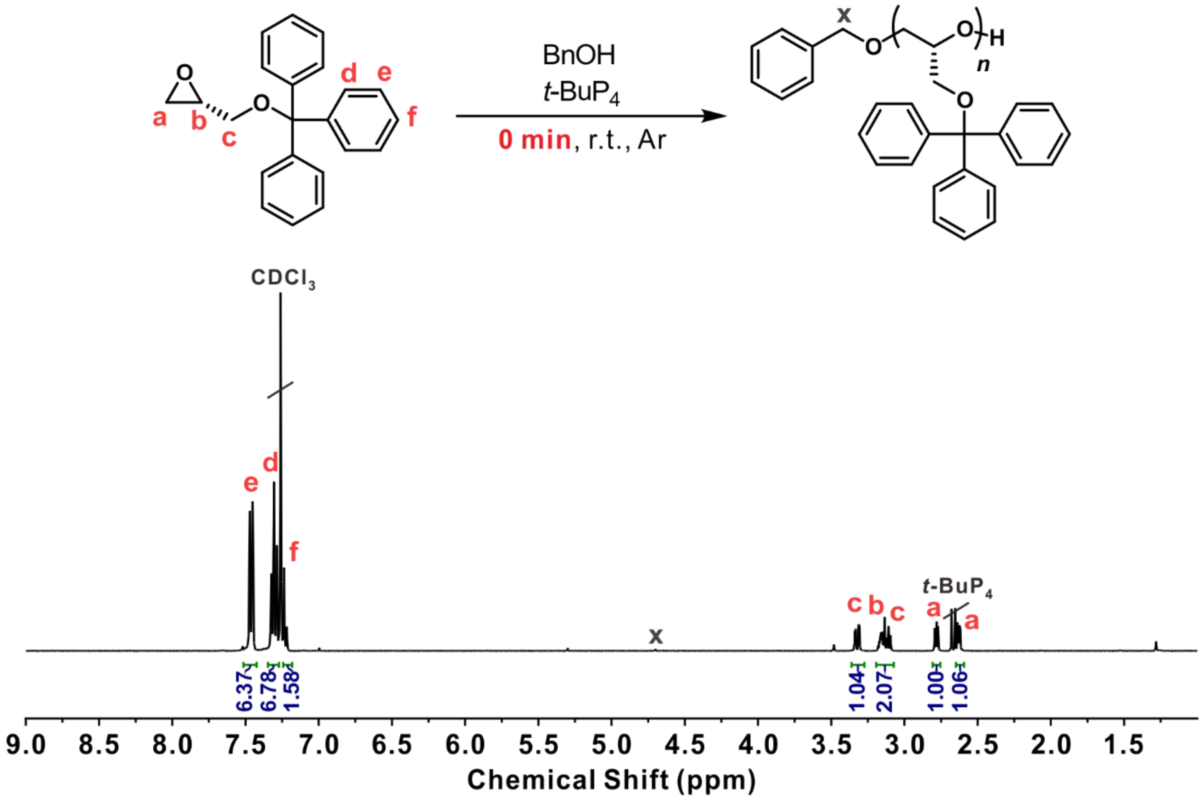

Supplementary Fig. 53. ^{1}H NMR spectrum of initial bulk polymerization of (*s*)-TGE monomer (400 MHz, $CDCl_3$): Conv. = 0.0%. Initial bulk polymerization occurred in the preparation of stainless jar containing reactants and three stainless-steel balls in glove box before performing ball milling reaction.

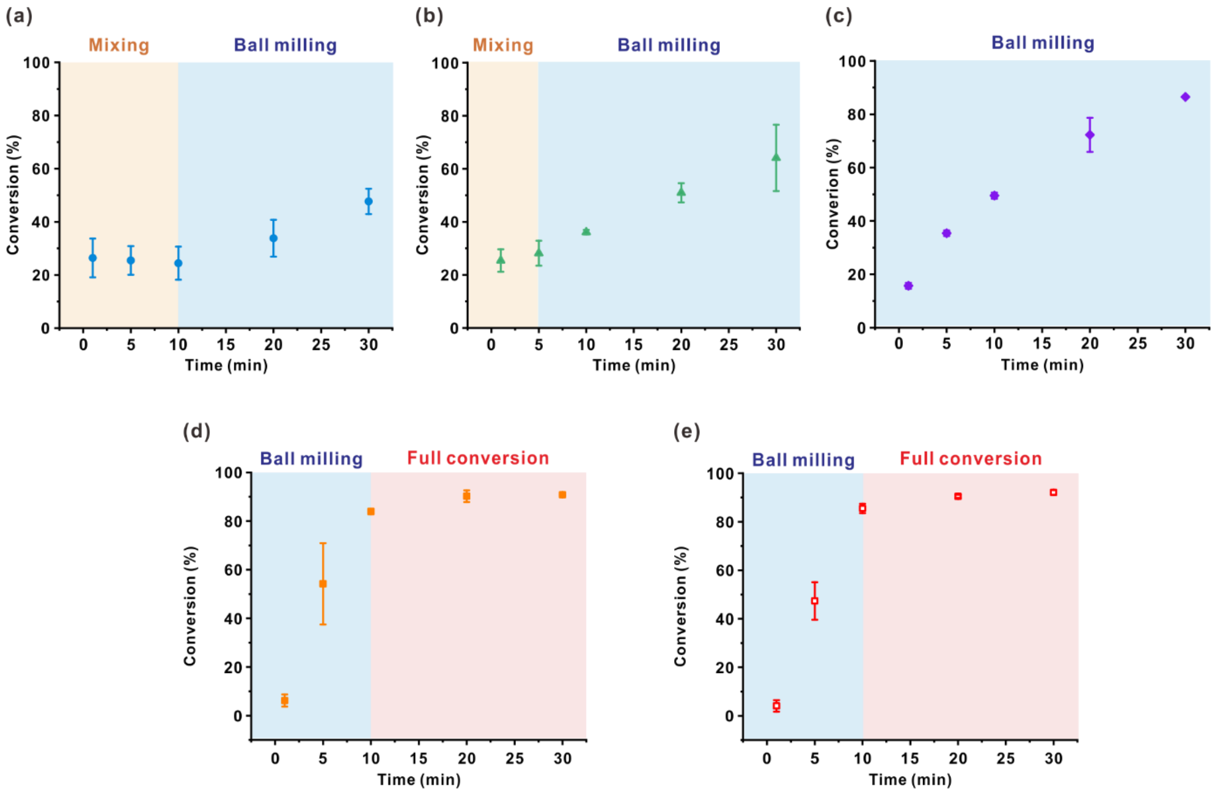

Supplementary Fig. 54. Type of the dominant reaction mechanism during ball milling polymerization of (a) PMPG, (b) PDPG, (c) PBPG, (d) PTGE, and (e) (*s*)-PTGE. In early reaction, the mixing process (yellow) is dominant in the case of less bulky monomers including MPG, and DPG unlike other bulky monomers. The ball milling process (blue) is gradually dominant as the reaction proceeded. Finally, the reaction process reaches a full conversion (red).

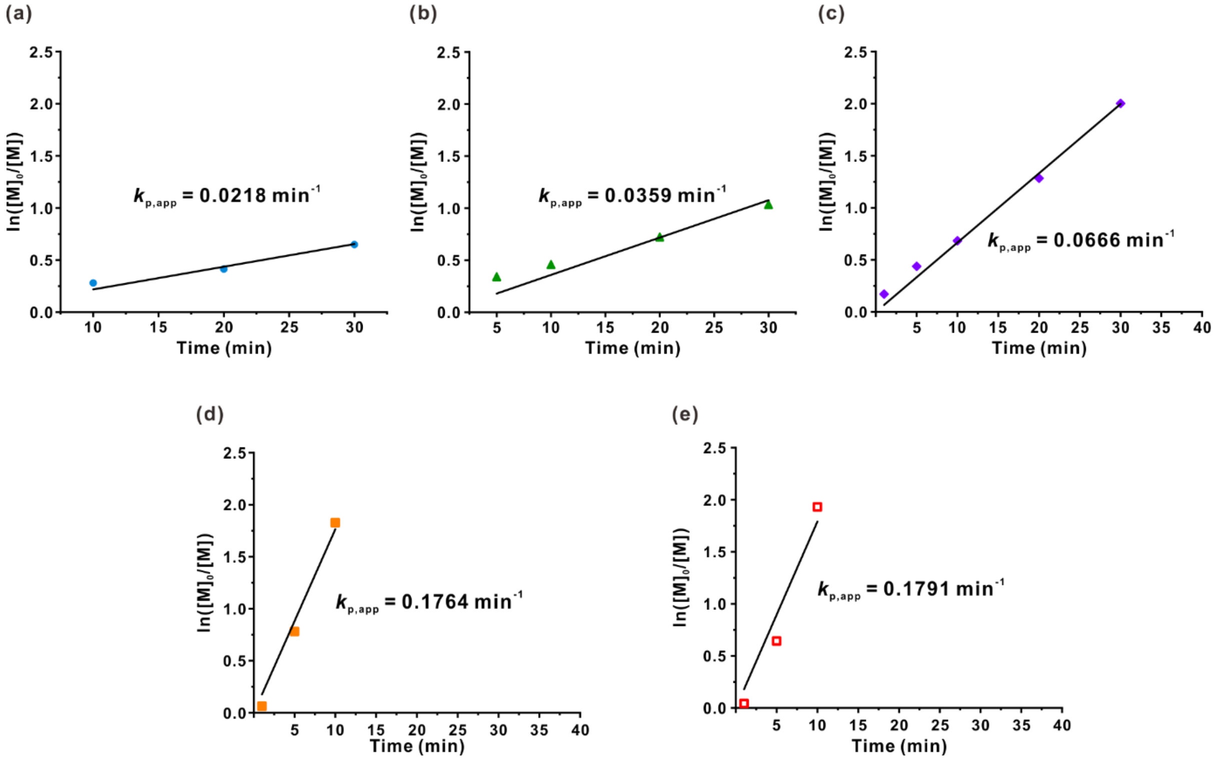

Supplementary Fig. 55. Polymerization kinetics of resulting polyether; First-order kinetic plot of ln([M]$_0$/[M]$_t$) over polymerization time of (a) PMPG, (b) PDPG, (c) PBPG, (d) PTGE, and (e) (*s*)-PTGE. The data corresponding to the ball milling mechanism were selected and plotted.

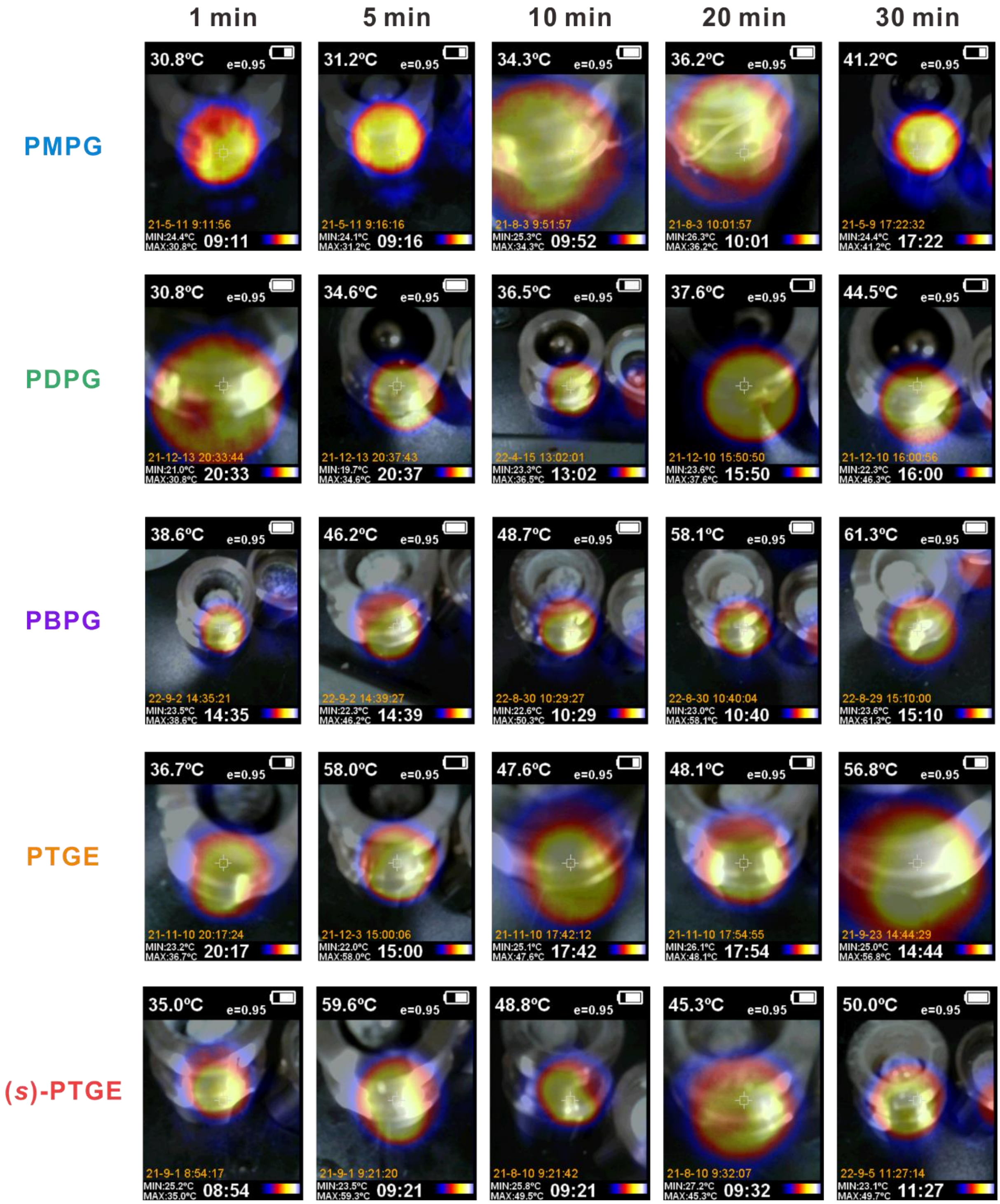

Supplementary Fig. 56. IR thermometer images showing temperature inside the jar after polymerization for each time at 30 Hz.

Supplementary Table 4. Comparison of monomer conversions under different experimental setup: ball milling, solution, and bulk polymerization.

Polymer	Conv.[a] (%)			
	Ball milling		Solution[b]	Bulk[c]
	ambient	w/ cooling jacket		
**PMPG**	47.7 ± 4.8	44.8	60.6	98.3
**PDPG**	64.1 ± 12.5	55.0	98.9	87.7
**PBPG**	86.5 ± 0.2	78.7	85.2	10.1
**PTGE**	90.8 ± 0.2	91.7	32.2	0
**(*s*)-PTGE**	92.1 ± 1.0	91.9	13.4	0

All polymerizations were targeted to a DP of 50 and conducted for 30 min. [a]Monomer conversion as calculated from the ^{1}H NMR spectrum of the crude monomer. [b]Solution polymerization in 2.5 M toluene at 60 °C. [c]Bulk polymerization at 60 °C.

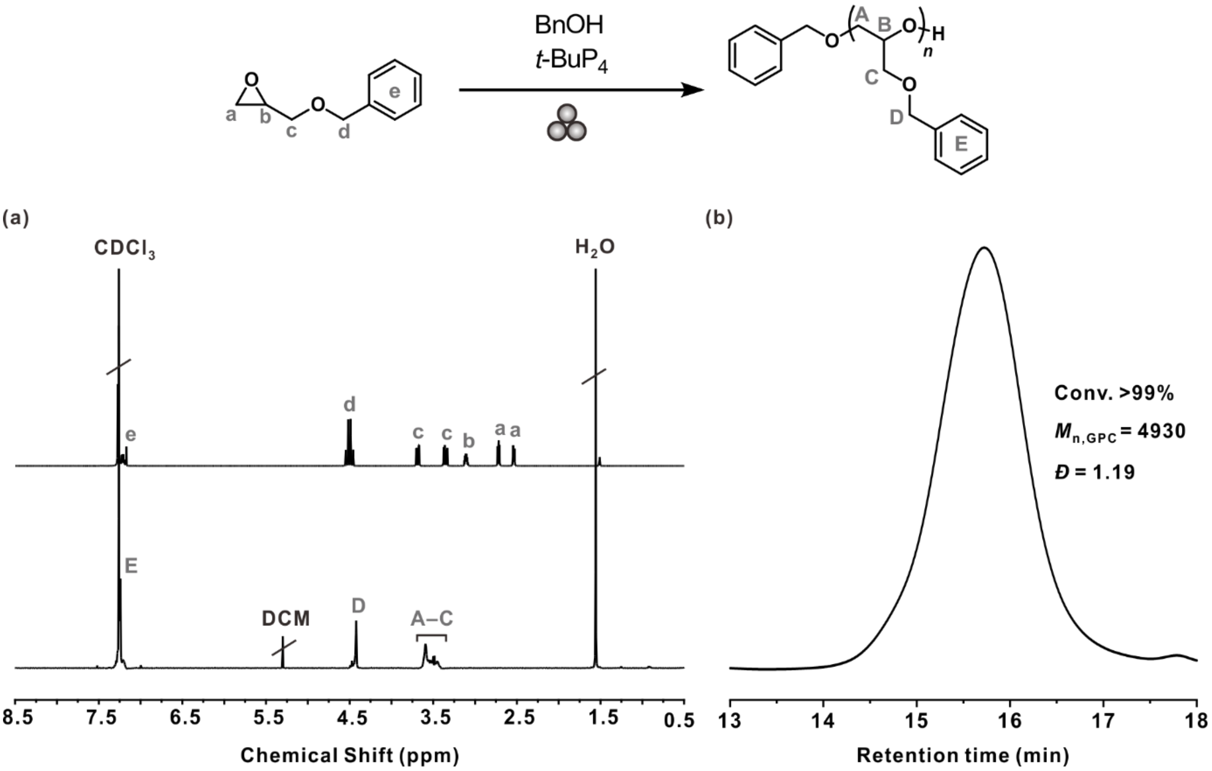

Supplementary Fig. 57. Ball milling AROP of liquid benzyl glycidyl ether (BGE) with a target DP of 50. A full conversion was observed in 20 min. (a) ^{1}H NMR spectrum (400 MHz, $CDCl_3$), and (b) GPC trace of the resulting poly(benzyl glycidyl ether) (PBGE).

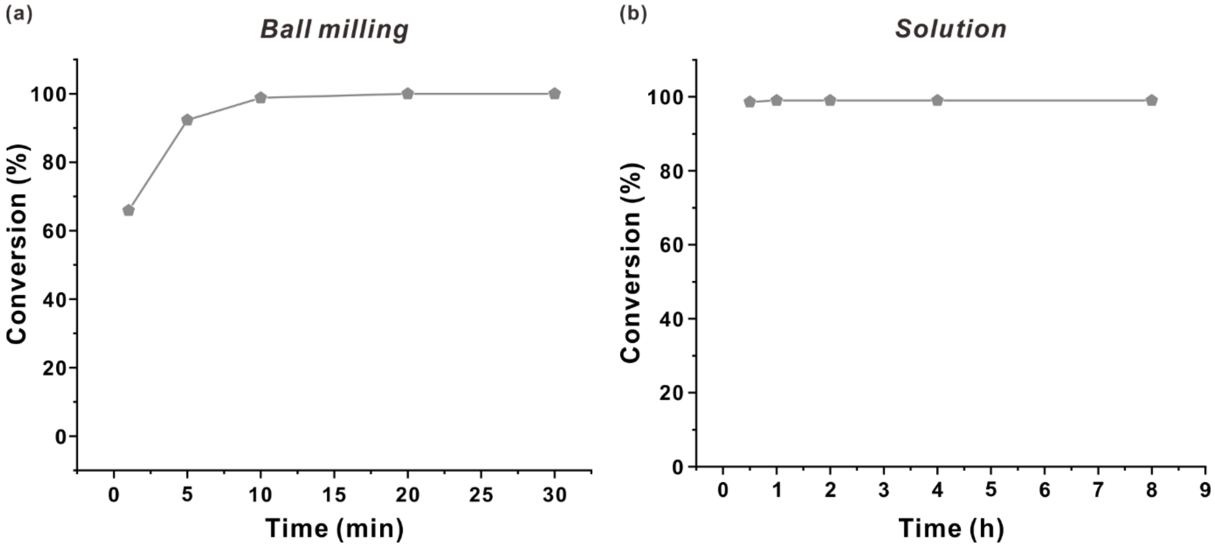

Supplementary Fig. 58. Comparative monomer conversions of the benzyl glycidyl ether (BGE) via polymerization under (a) solid-state ball milling polymerization, and (b) solution polymerization in toluene at 60 °C.

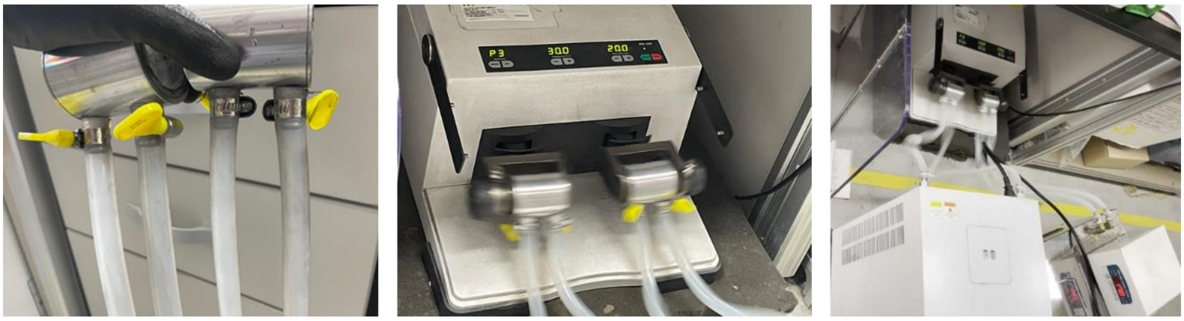

Supplementary Fig. 59. Temperature-controllable ball-milling equipment used in this study; cooling jackets (left), MM400 with cooling jackets (center), and overall mechanochemical polymerization setup with chiller (right).

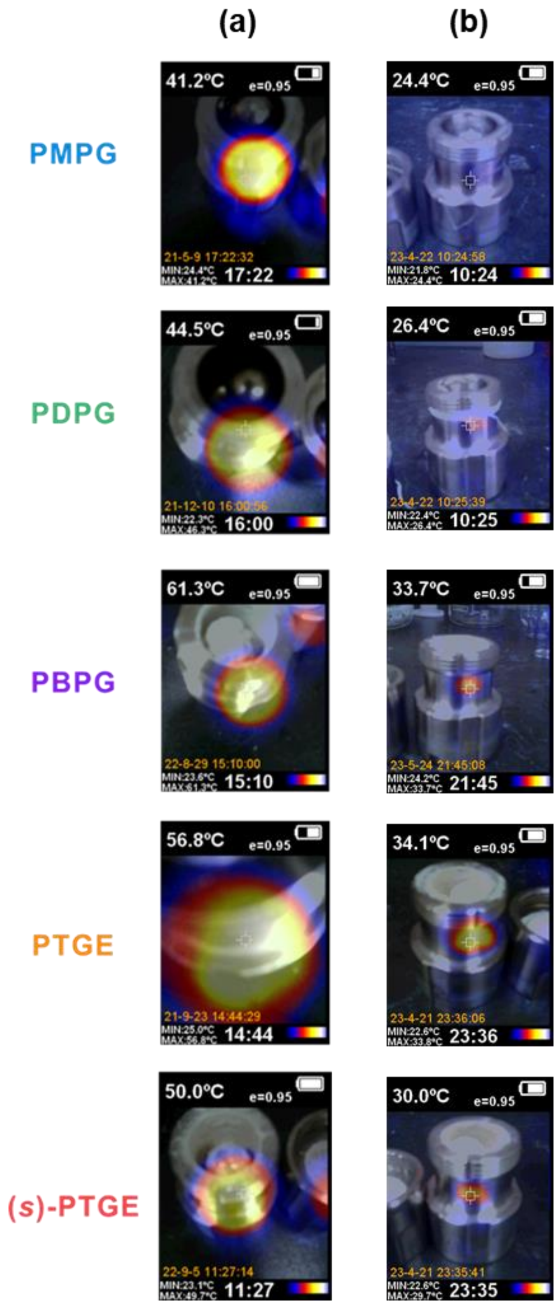

Supplementary Fig. 60. IR thermometer images showing temperature inside the jar after polymerization using (a) Ball-milling using MM400 under ambient condition and (b) temperature-controllable ball-milling MM400. All reactions were performed for 30 min at 30 Hz.

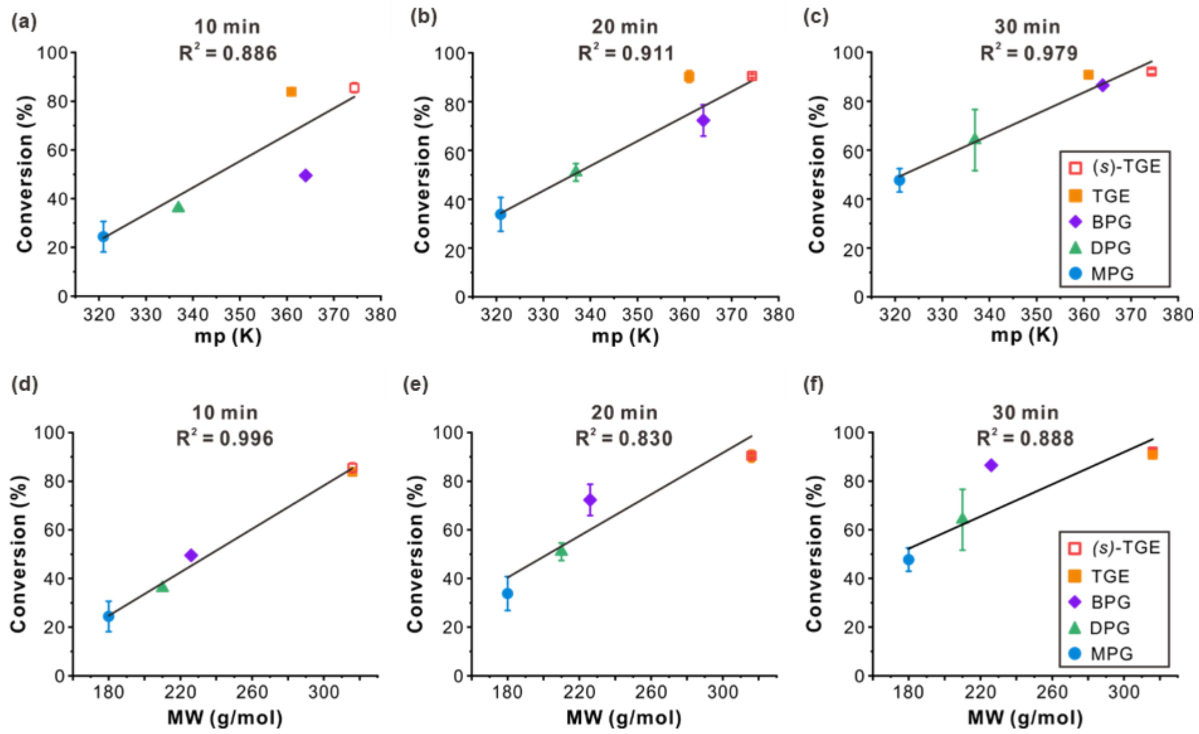

Supplementary Fig. 61. Series of plots of monomer conversion vs. melting point or molecular weight for the various functional epoxide monomers. (a–c) Series of plots of monomer conversion vs. melting point for various functional epoxide monomers at reaction times of (a) 10 min, (b) 20 min, and (c) 30 min, and (d–f) series of plots of monomer conversion vs. molecular weight for the various functional epoxide monomers at reaction times of (d) 10 min, (e) 20 min, and (f) 30 min. All data were collected in triplicate, and the average values were reported with standard deviation.

Supplementary Note 1. Mechanochemical polymerization conversion of (a) PMPG and PDPD, (b) PTGE and (s)-PTGE.

(a)

$$\text{Converion} = \frac{\int aromatic\ \text{H}\ in\ polymer}{\{\int aromatic\ \text{H}\ in\ polymer + (\int methylene\ \text{H}\ (a) in\ unreacted\ monomer * \#\ of\ aromatic\ \text{H})\}} * 100(\%)$$

(b)

$$\text{Conversion} = \frac{\int methylene\ backbone\ \text{H}\ in\ polymer}{[\int methylene\ backbone\ \text{H}\ in\ polymer + (\int methylene\ \text{H}\ (a)\ in\ unreacted\ monomer * 2)]} * 100(\%)$$

Supplementary Note 2. Theoretical molecular weight of polymer.

$$M_{n,th} = molecular\ weight\ of\ initiator + (molecular\ weight\ of\ monomer \times target\ DP)$$

Supplementary Note 3. Molecular weight of (a) PMPG and PDPG, (b) PTGE and (s)-PTGE calculated from ^{1}H NMR spectrum.

(a)

$$M_{NMR} = \left(\frac{\int aromatic\ proton\ in\ polymer}{\#\ of\ aromatic\ protons} * molecular\ wieght\ of\ monomer\right) + molecular\ weight\ of\ initiator$$

(b)

$$M_{NMR} = \left(\frac{\int methylene\ proton\ in\ polymer}{\#\ of\ methylene\ protons} * molecular\ weight\ of\ monomer\right) + molecular\ weight\ of\ initiator$$

Supplementary Note 4. Degree of polymerization (DP) of (a) PMPG and PDPG, (b) PTGE and (s)-PTGE calculated from ^{1}H NMR spectrum.

(a)

$$DP_{NMR} = \int aromatic\ proton\ in\ polymer/\#\ of\ aromatic\ protons$$

(b)

$$DP_{NMR} = \int (methylene\ proton\ in\ polymer/\#\ of\ methylene\ protons)$$

Supplementary Note 5. Initiation efficiency (IE%) calculated from MALDI-ToF spectrum at specific degree of polymerization (a) BnOH initiation and (b) self-initiation.

(a)

$$\mathrm{IE\%} = \frac{\int intiation\ from\ BnOH\ initiator}{(\int initiation\ from\ BnOH\ initiator + \int self - initiation)} * 100$$

(b)

$$\mathrm{IE\%} = \frac{\int self\ initiation}{(\int initiation\ from\ BnOH\ initiator + \int self - initiation)} * 100$$
